# Acknowledgement to Reviewers of the *International Journal of Molecular Science* in 2018

**DOI:** 10.3390/ijms20010232

**Published:** 2019-01-08

**Authors:** International Journal of Molecular Sciences Editorial Office

**Affiliations:** MDPI, St. Alban-Anlage 66, 4052 Basel, Switzerland

Rigorous peer-review is the corner-stone of high-quality academic publishing. The editorial team greatly appreciates the reviewers who contributed their knowledge and expertise to the journal’s editorial process over the past 12 months. In 2018, a total of 4095 papers were published in the journal, with a median time to first decision of 15 days and a median time to publication of 36 days. The editors would like to express their sincere gratitude to the following reviewers for their cooperation and dedication in 2018:
Aalen, Reidunn BirgittaLocatelli, MarcelloAasen, TrondLochhead, Jeffrey J.Abd El Hakim, YasminaLochman, JanAbdelrahman, MostafaLockwood, BrentAbdel-Sayed, PhilippeLoeken, Mary R.Abdel-Tawab, M.Loessner, DanielaAbejón, RicardoLoewendorf, Andrea I.Abenavoli, LudovicoLöffek, StefanieAbend, MichaelLognay, GeorgesAbeywardana, TharindumalaLoh, KimAbou Neel, Ensanya A.Lohoff, FalkAbraham, DietmarLoinard, CélineAbraham, EleniLoiselle, Denis S.Abu-Romman, SaeidLombardi, RaffaellaAbuzaid, AhmedLonardo, AmedeoAcheva, AnnaLondei, PaolaAchinivu, EzinneLong, YichengAcosta Contreras, Florentina NiurisLonghi, SoniaAcosta-Motos, Jose RamónŁoniewski, IgorAcuña-Castroviejo, DaríoLönnberg, TuomasAda, PopoloLooijenga, Leendert H. J.Adam, VojtěchLopaschuk, Gary D.Adamakis, Ioannis-DimosthenisLópez, ConcepciónAdamek, AgnieszkaLópez, JoséAdams, JamesLópez, María L.Adhikary, AmitavaLopez, MiguelAdriaensens, Peter J.López-Alonso, DiegoAdunyah, Samuel E.Lopez-Camarillo, CesarAeddula, Narothama ReddyLopez-Escamez, Jose A.Afrin, RehanaLopez-Ferber, MiguelAfrin, SadiaLopez-Llorca, Luis V.Agarwal, SumitLopez-Moya, FedericoAgatemor, ChristianLópez-Tirado, JavierAgeitos, Jose ManuelLopez-Viñas, EduardoAgerbirk, NielsLoPiero, AngelaAggarwal, AbhishekLopresti, PatriziaAgnelli, LucaLora, JorgeAgnieszka, SzopaLorber, AvrahamAgnoli, KirstyLorberboum-Galski, HayaAgostini, MarcoLorens, James B.Agrawal, PrashansaLorenz, KristinaAgrawal, SwatiLorenzo González, ÓscarAguilar, Ludwig ErikLorenzo, MicheleAgut, MontserratLorico, AurelioAhlenstiel, ChantelleLosano, GianniAhluwalia, AmritaLosurdo, GiuseppeAhmad, AamirŁotocka, BarbaraAhmad, EjazLou, SongAhmad, ShakilLoudig, Olivier D.Ahmed, ChulbulLoutradis, DimitriosAhmed, HafizLouveau, IsabelleAhmed, Mahmoud S.Loveridge, JoelAhmed, MaryaLowe, Julie M.Ahn, Jee-YinLu, Jeng-WeiAhvazi, BehzadLu, JingAiello, FrancescaLu, JunAkamatsu, WadoLu, LinchaoAkhavan, OmidLu, Mei-ChinAkiba, YasutadaLu, QuanlongAkimov, Sergey A.Lu, Shao-ChunAkıncılar, Semih CanLu, ShaoyongAkita, SadanoriLu, StevenAkitsu, TakashiroLu, Wei-YuAkkuratov, E. E.Lu, YouAlaimo, AlessandroLu, ZhanpingAlam, Mohammad ParvezLuborsky, Judith L.Alam, ShafiulLucas-Barbosa, DaniAlapan, YunusLucek, KayAlba, JuanLucena, María IsabelAlbano, EmanueleLuchetti, AndreaAlbrecht, Eric A.Luciani, GiuseppinaAlbrecht, RandyLuciano, Alberto MariaAlcaraz, Maria JoséLucini, CarlaAlder, JanetLúcio, MarleneAldred, KatieLuco, Reini F.Aldred, NicholasLuculescu, Catalin RomeoAl-Dujaili, EmadLuczak, MagdalenaAleksandrov, AlexeyLudueña, RichardAleman, Ignacio TorresLudwiczuk, AgnieszkaAlexaki, Vassilia IsminiLugnier, ClaireAlexander, H. DenisLühder, FredAlexander, MathewLuís, ÂngeloAlexander, RebeccaLukac, PavelAlexiou, ChristophLukas, JanAlfano, MassimoŁukasik, RafałAlfaro-Moreno, ErnestoLumniczky, KatalinAlfarouk, KhalidLuna, AurelioAlferink, JudithLund, JennyAlfieri, AndreaLund, LarsAlfieri, CarloLundholm, LovisaAlfonsi, RominaLundstrom, KennethAl-Horani, RamiLuo, Kai HongAli, AzaharLuo, MengAlibakhshi, Mohammad AminLuo, RayAlicia, PickrellLuo, RongcongAliev, GjumrakchLuo, XuAllen, AntinoLuo, ZonghuaAllen, CaitilynLuongo, LivioAllen, RandyLupiañez, José AntonioAllione, AlessandraLupini, AntonioAllmang, ChristineLupo, GiuseppeAlmasan, AlexLuppi, FabrizioAl-Mashhadi, Rozh H.Luque, FranciscoAlmed, NuzhatLusczek, Elizabeth R.Almeida, HenriqueLushchekina, SofyaAlmeida, Luciana OliveiraLütjohann, DieterAlmeida, PedroLuvisetto, SiroAlmeida, Rodrigo C.Lyle, Alicia N.Almodóvar, JorgeLymperopoulos, AnastasiosAlmstrup, KristianLyng, HeidiAlonso, LeocadioLyu, HailongAlonso-Magdalena, PalomaLyu, HuiAlpini, GianfrancoLyu, YuanzhiAlpizar, Yeranddy A.Lyubchenko, YuriAlqudah, AhmedM’Koma, Amosy E.Altomare, ClaudiaMa, GuojiaAlvarez, CarlosMa, LiangAlvarez, Juan B.Ma, Wen-JuanÁlvarez-Sánchez, NuriaMa, XingAlvarez-Suarez, JoséMa, XingmaoAlves, ElianaMa, Zheng FeeiAlves, SandraMaak, SteffenAlzaid, F.Mabuchi, MamoruAmadoro, GiuseppinaMacabeo, Allan P.Amanda, NorvellMacauley, Matthew S.Amar, LaurenceMaccallini, CristinaAmaral, AlexandraMacchi, BeatriceAmarowicz, RyszardMacdonald, AndrewAmbati, JayakrishnaMacDonald, Clinton C.Ambrosio, Maria RaffaellaMacedo, FatimaAmeri, PietroMacedo, M. PaulaAmeye, MaartenMachado, Mariana V.Ami, DilettaMachado, RicardoAmillis, SotirisMacias, Vanessa M.Amini-Nik, SaeidMacias-Gonzalez, ManuelAmirkhani, MasoumeMaciejczyk, MateuszAmital, HowardMaciejewska, DominikaAmmendola, MicheleMacín, Stella MarisAmmendola, RosarioMack, Andreas F.Amorós, AsunciónMackereth, CameronAmpofo, EmmanuelMacLean, Andrew G.Amrani, YassineMacmillan, Colleen P.Amstislavsky, SergeiMadala, Hanumantha RaoAn, YuMaddelein, Marie-LiseAnandhan, AnnaduraiMaddineni, PrabhavathiAnanieva, KalinaMADEDDU, Roberto BeniaminoAnastasiadou, MariaMadej, M. GregorAncona-Contreras, VeronicaMadesis, PanagiotisAncuceanu, RobertMadine, JillianAndersen, CatherineMadureira, Patrícia A.Anderson, Mark T.Maeda, AkikoAnderson, Sarah N.Maeda, KazuhisaAndjelkovic, Anuska V.Maeda, TomokiAndrade, ArturoMaffei, AngeloAndrade, Paula B.Maga, GiovanniAndre, HelderMaggi, DavideAndreadou, IoannaMaggini, RitaAndreasson, ErikMaggiolini, MarcelloAndreev, KonstantinMagherini, FrancescaAndres, Allen M.Magiatis, ProkopiosAndressen, KjetilMagierowski, MarcinAndrisani, Ourania M.Magini, AlessandroAng, WeeMagnaghi, ValerioAngel, PeggiMagnaldo, ThierryAngelino, DonatoMagnani, MauroAngelone, TommasoMagni, FulvioAngeloni, ChristinaMagnusson, KarinAngelova, AngelinaMagnusson, Karl-EricAngelova, MihaelaMahalingam, RaviAngelucci, AdrianoMahavadi, PoornimaAngermann, JeffMaher, PamelaAniello, FrancescoMahmood, KhalidAnifandis, GeorgeMai, StefaniaAnisimova, IrinaMaida, YoshikoAnji, AntjeMaier, PatrickAnken, RalfMaiorano, DomenicoAnower, RokebulMaisch, TimAnraku, MakotoMajeed, WaqarAnsa, BenjaminMajhen, DragomiraAnsaldi, MireilleMajhi, PrabinAntognelli, CinziaMajlessi, LalehAnton, SonnenbergMajor, IanAntonangeli, FabrizioMajtan, JurajAntoniadi, IoannaMajumdar, SoumyajitAntonio Speretto, RaulMajumder, MrinmoyeeAntonova, Ekaterina PetrovnaMakarevich, PavelAntony, JaneMakarevitch, IrinaAnwar, RokebulMakena, MonishAoki, DanMakieva, SofiaAoki, KazuhiroMakino, HiroshiAparicio Blanco, JuanMakishima, MakotoApone, FabioMakiyama, TakeruAppell, MichaelMakvandi, PooyanArai, KenMalacrinò, AntoninoArai, ToshiroMalaguarnera, GiuliaAran, GemmaMalara, AlessandroArana, AnaMalard, VeroniqueAraniciu, CătălinMalavolta, MarcoArboleda, Valerie A.Malde, AlpeshArchacki, RafalMalekigorji, MaryamArdelean, Lavinia CosminaMalemud, CharlesArdley, JulieMalemud, Charles J.Aref, Amir RezaMalerba, DonatoArena, SabrinaMalerba, GiovanniArevalo, Juan CarlosMali, VishalArgmann, Carmen A.Malik, RavinderArguelles, SandroMallamace, FrancescoArgüello, José M.Malle, ErnstArianna, MaruccoMallein-gerin, FrédéricAriga, KatsuhikoMallipeddi, Prema LathaArimura, ShinichiMalmberg, PerArino, JoaquinMalpeli, GiorgioArispe, NelsonMaltesen, RalucaAriyoshi, WataruMalvolti, Maria EmiliaAriza, Maria EugeniaMammola, Caterina LoredanaArmand, MartineManara, AnnaArmani, AndreaMaňásková-Postlerová, PavlaArmanini, DecioManavalan, BalachandranArmentano, IlariaManca, Maria LetiziaArmstrong, MichelleMancini, CeciliaArndt, Patrick G.Mancini, Stéphane J. C.Arnesano, FabioMancone, CarmineArnould, ThierryMancuso, AndreaArora, GunjanMancuso, CesareArtyszak, ArkadiuszMandard, StéphaneAryal, RishiMandato, ClaudiaAryal, Uma K.Mandava, NandaAsa, SylviaMandrioli, MauroAsahi, MichioManga, PrashielaAsahina, KinjiMangalagiu, IonelAsai, AkiraManini, Mhd LouaiAsakura, TomikoManjunath, ManuboluASANO, RyutaroManley, Sharon J.Asbell, Penny A.Manner, SuviAscoli Marchetti, AndreaMannik, JaanAshida, HitoshiManninger, MartinAskarian-Amiri, Marjan E.Manocha, GunjanAskes, SvenMånsson, AlfAslam, MuhammadMante, Francis K.Aspatwar, AshokManthey, John A.Aspenström, PontusManuelli, MatteoAssirelli, ElisaManzo, CiroAstegno, AlessandraManzoor, RashidAtamian, HagopMao, Xiao WenAterini, StefanoMapelli, LisaAtkinson, John P.Marasco, DanielaAtobe, MasakazuMarc, GabrielAtsuta, IkiruMarcato, PaolaAttanasio, ChiaraMarceau, FrançoisAttar, HooyarMarchant, Jonathan S.Attarian, ShahramMarchesan, SilviaAtteritano, MarcoMarchetti, MartaAuberger, PatrickMarci, RobertoAudet, CélineMarco, FranciscoAunapuu, MarinaMarco-Contelles, JoséAushev, VasilyMarcovici, GenoAustin, CarolineMarcuzzi, AnnalisaAvanesyan, AlinaMarczak, MałgorzataAvci-Adali, MeltemMarechal, AlexandreAvila, JesúsMarechal-Drouard, LaurenceAvila-Román, JavierMarengo, BarbaraAvolio, AlbertMargalef Jornet, MariaAw, KeanMargaria, PaoloAwayda, MouhamedMargaritis, LukasAxpe, Enekomargherita, maioliAydemir, TolunayMargit, BalázsAyers, DuncanMari, MicheleAyyar, Balasubramanium VijayalakshmiMaria José, Rodriguez LagunasAzarnia Tehran, DomenicoMaria, Naomi I.Azer, Samy A.Márialigeti, KárolyAzevedo, HerlanderMariconti, MaraAzevedo, Nuno F.Mariggiò, Maria A.Azizi, EbrahimMarigo, IlariaAzmi, Asfar S.Marin, Daniela ElizaAzuma, MizukiMarin, José Juan GarcíaAzuma, YoshiakiMarincs, FerencAzushima, KengoMariniello, LoredanaB. Nasrallah, JuneMarino, JosephBaas, PaulMarino, TizianaBaba, YuhMariot, VirginieBabcock, DanielMariotto, SofiaBabu, AnishMarkiv, AnatoliyBabu, R. JayachandraMarklund, UlrikaBaby, ShineMarkopoulou, OlgaBacelar, EuniceMarla, SandeepBach, Duc-HiepMarminon, ChristelleBack, KyoungwhanMaroja, Luana S.Bäck, MagnusMarone, ElettraBäck, NilsMaroni, PaolaBackert, SteffenMaroteaux, LucBácskay, IldikóMarqués Villavecchia, Ana M.Bączek, TomaszMarques, NatáliaBaczek-Kwinta, RenataMarrs, James A.Badawi, MohamedMarschall, Hanns-UlrichBado, IgorMartel, FátimaBadylak, Stephen F.Martel, PauloBae, Hae-RahnMartí, SergioBae, HyunsuMartí-Calatayud, Manuel CésarBae, Ki HyunMartin, AbrahamBae, Ok-NamMartin, Andrew V.Bae, SoochanMartin, Chelsea K.Baenas, NievesMartin, FinianBaer, Patrick C.Martin, FranciscoBag, SudeepMartin, IanBagavant, HariniMartín, Juan FranciscoBaghdadi, Ziad D.Martin, LaetitiaBagnoli, PaolaMartin, Luc J.Bai, DonglinMartin, PatriciaBai, GangMartin, WilliamBai, HuaMartín-Antonio, BeatrizBai, RenrenMartín-Aragón, SagrarioBai, YunMartin-chouly, CorinneBaird, NicholasMartinelli, PaolaBajda, MarekMartínez De Paz, Pedro JoséBajpai, RichaMartínez Iglesias, OlaiaBak, IstvanMartínez, AlbertoBaker, YsobelMartinez, AnaBakht, MartinMartinez, Anne-sophieBakhti, MostafaMartínez, AntonioBalabanian, KarlMartínez, Luis CarlosBalasubramaniam, MeenakshisundaramMartinez, ManuelBalcerczyk, AnetaMartínez-Cañamero, MagdalenaBaldanzi, GianlucaMartínez-Contreras, Rebeca D.Baldisserotto, AnnaMartínez-Esteso, María JoséBaldovino, SimoneMartínez-Gómez, PedroBaldrich, PatriciaMartinez-Lostao, LuisBalestrazzi, AlmaMartinez-Sanchez, AidaBalestrieri, EmanuelaMartínez-Soler, FinaBali, ShilpaMartinez-Useros, JavierBall, JonathanMartin-Fontecha, AlfonsoBall, K. AureliaMartín-Hernández, DavidBallerini, PatriziaMartini, ClaudiaBallizany, WouterMartini, DanielaBallmer-Hofer, KurtMartini, MaurizioBalloy, VivianeMartino, SabataBally, JuliaMartin-Pena, AlfonsoBally, MartaMartins, AlbinoBalmayor, Ph.D., Elizabeth R.Martins, Ian JamesBaltriukiene, DaivaMartins, IsabelBaluska, FrantisekMartins, Maria RosarioBambling, MatthewMartins, NatáliaBan, YusukeMartins, Sandra Fátima FernandesBanci, LuciaMartinvalet, DenisBandini, ErikaMartín-Vasallo, PabloBanerjee, AditiMartorana, AlessandroBanerjee, Hirendra NathMartucciello, StefaniaBanerjee, KalpitaMaruyama, IchiroBanerjee, ParthaMaruyama, KeiBanerjee, Sushanta K.Marverti, GaetanoBanfalvi, ZsofiaMary Ellen, UrickBanfi, LucaMarycz, KrzysztofBangoura, BeritMarzaro, GiovanniBanilas, GeorgiosMarzocco, StefaniaBanning, AntjeMarzullo, PaoloBanno, KoujiMas, EricBano, DanieleMasaki, TsutomuBanobre, ManuelMasalova, Olga V.Banoub, JosephMasarik, MichalBanti, Christina N.Masauzi, NobuoBanys-Paluchowski, MalgorzataMaserti, Bianca ElenaBao, Yong-PingMashima, RyuichiBao, YupingMasi, AntonioBarabas, PeterMasieri, FedericaBarakat, KhaledMasli, SharmilaBaralle, MarcoMasłyk, MaciejBaran, MarzenaMassarotti, AlbertoBaranello, LauraMassey, Andrew J.Baranov, VladislavMassey, StevenBaranowska-Bosiacka, IrenaMásson, MárBararpour, TaghiMassotte, DominiqueBarata, JoãoMassoumi, RaminBarault, LudovicMastrangelo, Anna M.Barbagallo, DavideMasuda, JunkoBarbaro, BarbaraMasuda, KiyoshiBarbeck, MikeMasui, KentaBarber, Paul RichardMasullo, MariorosarioBarberi, TizianoMatarrese, PaolaBarbero, AndreaMatchkov, VladimirBarbier, François F.Mateo, CesarBarbieri, ElenaMatesanz, Ana I.Barbieri, FedericaMateusz, MateuszBarbieri, Silvia S.Mathuru, Ajay S.Barbillon, GrégoryMati-Baouche, NarimaneBarbolina, MariaMatoba, NobuyukiBarbosa, Flávia LeãoMatrisian, Lynn M.Barbosa, Judite N.Matsubara, KeiichiBarcelo, JuanMatsuda, MasaruBarchetta, IlariaMatsugaki, AiraBardet, MichelMatsugo, SeiichiBardi, GiuseppeMatsui, ToruBardy, CedricMatsumoto, Ken'ichiroBaregamian, NairaMatsumoto, KunioBarile, LucioMatsumoto, TaroBarillari, GiovanniMatsumoto, YasuhikoBarker, Gareth R.Matsunami, KatsuyoshiBarla, ThirupathiMatsuo, MuneakiBarman, ApurbaMatsuoka, IsaoBarnard, NealMatsuoka, MasatoBarone, PaoloMatsushita, TakehikoBarone, RitaMatsuura, BunzoBarral, DuarteMatsuzaka, KenichiBarrero, Maria J.Matsuzaki, GoroBarrio, RosaMatta, CsabaBarros, FranciscoMatta, JaimeBarros, Pedro M.Mattei, FabrizioBarrott, JaredMattijssen, FritsBar-Shavit, ZviMattoscio, DomenicoBarta, CsengeleMatucci Cerinic, MarcoBartelt, AlexanderMatusica, DusanBartolazzi, ArmandoMatwijczuk, ArkadiuszBasak, DebasishMaugeri, AndreaBasak, SujitMaugeri, MarcoBasco, Leonardo K.Mauk, Michael G.Bascom, GavinMaury, EleonoreBashkin, James K.Maurya, ShailendraBasini, GiuseppinaMavian, CarlaBasit, AbdulMavridis, IoannisBaslam, MarouaneMavridis, KonstantinosBasoli, ValentinaMavrogonatou, EleniBassareo, Pier PaoloMaxwell, AnthonyBasso, DanielaMayanil, ChandraBastarrachea, Luis J.Mayes, SeanBasu, AlakanandaMayne, ChristopherBasu, SwarajMayo, KevinBatarseh, YazanMayor, David F.Bates, John T.Mayorov, AlexanderBathula, ChandraMayr, ChristianBatsché, EricMazars, ChristianBattaglia, EricMazerska, ZofiaBattaglia, FortunatoMazor, OhadBattista, SabrinaMazur-Bialy, Agnieszka IrenaBattistini, LuciaMcArdle, StephanieBauckneht, MatteoMcAuley, Julie L.Bauer, Andrew JohannMcBride, Devin W.Baues, ChristianMcCarthy, ElizabethBaulch, JanetMcCaughan, Geoffrey WilliamBaumstark-Khan, ChristaMcClelland, SarahBawadekar, MandarMcCoard, SueBax, BenMcCormick, CraigBayat, VafaMcCubbin, AndrewBayer, PhilippMcDaneld, Tara G.Bayfield, MarkMcDermott, Suzanne M.Bayik, DefneMcDonald, AllisonBayin, N. SumruMcDonald, CourtneyBazire, AlexisMcDougal, Owen M.Bazylińska, UrszulaMcDougall, GordonBazzigaluppi, PaoloMcDowell, AndrewBeberashvili, IliaMcErlean, PeterBeberok, ArturMcFeeters, RobertBeccano-Kelly, DayneMcGarry, Roisin C.Becker, Therese M.McGowan, EileenBécuwe, PhilippeMcGowan, SheenaBednarek, PiotrMcGregor, AlistairBeharry, Kay D.McGregor, CeciliaBehera, SmrutisanjitaMcInerney, GeraldBeilby, MaryMcKay, BrianBeis, DimitrisMcKee, Lauren S.Beitz, EricMcMacken, GraceBekeschus, SanderMcMillin, MatthewBekri, SoumeyaMcMullin, David R.Belayew, AlexandraMeabon, James S.Belenguer, AlvaroMedeiros-Domingo, ArgeliaBelfrage, Anna KarinMedina Piles, VicenteBelitsky, Jason M.Medina, Francisco JavierBell, StephenMedina, MiguelBellavia, DanieleMedina, Reinhold J.Bellen, Hugo J.Medová, MichaelaBellosta, PaolaMeesters, DennisBellucci, AriannaMehedi, MasfiqueBeloor, JagadishMehla, JogenderBełtowski, JerzyMeier, Raphäel P. H.Bencsik, PeterMeierhofer, DavidBendahhou, SaïdMeijer, HJGBendas, GerdMeijer, OnnoBenedetti, MicheleMeijles, Daniel N.Benesic, AndreasMejuto, Juan CarlosBenhadou, FaridaMela, PetraBenhalima, KatrienMelagraki, GeorgiaBenini, StefanoMelchor, JuanBenistant, ChristineMeldolesi, JacopoBenjamin, DonMele, LauraBennett, James P.Meli, AlbanoBentel, Jacqueline M.Mellody, K. T.Berardi, AnnaMelnikov, StanislavBerdis, Anthony J.Melnyk, CharlesBerent-Maoz, BeataMelo, Ana M. P.Berenyiova, AndreaMeloni, BrunoBeretta, GiovanniMeloni, GabrieleBergamo, AlbertaMemarzadeh, KavehBergdahl, AndreasMenasché, PhilippeBerger, WalterMenassa, RimaBergero, RobertaMendes, MateusBergler, HelmutMendez, JuanBergonzi, SaraMéndez, LucíaBerillis, PanagiotisMéndez-Álvarez, EstefaníaBerindan-Neagoe, IoanaMendez-Sanchez, NahunBerkowitz, Bruce A.Mendola, Diego La Berlanga, Ángel MéridaMendonca, AntonioBerlin, K. DarrellMendonça, NunoBermudez, MarcelMenendez, EstherBernacchioni, CaterinaMeng, DongBernal, M. MarMeng, HaoBernard, Bruno A.Meng, RanBernardi, HenriMeng, WeihuaBernardo, LetiziaMeng, XiaoliBernardo, UmbertoMeng, Yuan XiangBernitsas, EvanthiaMenon, ManojBernstein, Audrey M.Menu, ElineBerta, GraziellaMerah, OthmaneBertea, CinziaMeraviglia, VivianaBerthelot, LaurelineMercati, FrancescoBerthod, FrançoisMercer, CarolBertinaria, MassimoMercier, CorinneBertoli, GloriaMereu, ElisabettaBertrand, LucMerino, Jaime M.Berzal-Herranz, AlfredoMerino, RamónBessa, LucindaMerlino, AntonelloBessette, BarbaraMerlo, GiorgioBesson, ThierryMerret, RémyBetekhtin, AlexanderMeruvu, SunithaBetemariam, Senait AshenafiMerzendorfer, HansBethke, GeritMesaros, Clementina A.Bettaieb, AliMessa, PiergiorgioBevan, DavidMessaritakis, IppokratisBevington, AlanMessina, Concetta MariaBeyer, SebastianMessini, Christina I.Bhalla, VivekMessori, LuigiBharadwaj, VivekMetharom, PatBharti, KapilMetzinger, LaurentBhatia, Harsharan S.Meulia, TeaBhatta, MadhavMeyer, GregoryBhattacharjee, SonaliMeyerowitz, ElliotBhattacharya, RajatMezzasalma, ValerioBhave, DevayaniMicek, AgnieszkaBhavsar, Amit P.Micevych, PaulBhullar, Rajinder P.Michaelides, Sozos N.Bhusal, Siddhi JeewanMichalak, IzabelaBiagi, MarcoMichalik, JanBiagini, GiuseppeMicheau, OlivierBiamonti, GiuseppeMicheletti, GabrieleBianchi, GiuseppeMichelhaugh, Sharon KayBianco, PieroMicillo, RaffaellaBiase, FernandoMiele, Adriana E.Biasini, EmilianoMiele, ClaudiaBiasucci, GiacomoMielenz, DirkBichet, Daniel G.Mierke, Claudia TanjaBidwai, AshokMiernyk, Ján A.Biedermann, DavidMignatti, PaoloBiedermann, ThomasMiguel, SilviaBiernasiuk, AnnaMiguel, Sónia P.Biersack, BernhardMihranyan, AlbertBiffis, AndreaMijailovich, SrbaBigiani, AlbertinoMikami, KojiBijak, MichałMikecz, KatalinBijlsma, Maarten F.Miki, DaisukeBilleter, AdrianMiki, YasuhiroBilodeau, SteveMiklossy, GabriellaBirch, JohnMikropoulos, ChristosBirchler, James A.Mikstacka, RenataBird, AmandaMilanesi, AnnaBirk, UdoMilano, FrancescoBirman, SergeMilano, GiuseppinaBironaité, DaivaMilcovich, GesmiBisaga, MaciejMiles, Wayne OwenBishehsari, FarazMiletta, MariaBisignano, GiuseppeMilhiet, Pierre-EmmanuelBisio, AlessandraMilia, EgleBišová, KateřinaMiller, Alexandra C.Bisti, SilviaMiller, CharlesBiswas, SangitaMiller, EdmundBiswas, SantanuMiller, GalenBiswas-Fiss, Esther E.Miller, JustinBitler, BenjaminMiller, MarkBitonti, Maria B.Millet, OscarBittel, Douglas C.Millhauser, GlennBizzarri, MarianoMills, KenBjörnsson, EinarMilojevic, TetyanaBlacker, ThomasMilosavljevic, NinaBlagden, SarahMilovanovic, DragomirBlais, AnneMin, Kyung-JinBlandino, G.Minamoto, ToshinariBlank, VolkerMinarchick, ValerieBlankesteijn, W. MatthijsMinarovits, JanosBlanton, CynthiaMinato, Ken-ichiroBlasi, JuanMinde, DavidBlasiak, JanuszMine, YuichiBłasiak, JanuszMinervini, GiovanniBlein-Nicolas, MélisandeMinghetti, LuisaBlock, AnnaMinkiewicz, PiotrBlokland, ArjanMinnerup, JensBlomme, EricMino, MasanobuBlomme, JonasMinocha, Subhash C.Blonska, MarzennaMinucci, SergioBlume-Jensen, PeterMiozzi, LauraBlumenberg, MiroslavMiranda Salvado, Isabel MargaridaBlumenthal, EdwardMiranda, CristobalBoag, PeterMircheff, Austin K.Bobe, RégisMirete, SalvadorBocianowski, JanMirey, GladysBock, Karl WalterMiri, Amir K.Bocos, CarlosMirzayans, RazmikBoda, EnricaMirzayanz, RazmikBode, BarrieMisawa, KiyoshiBodenstine, ThomasMishin, Alexander S.Bodily, JasonMishina, MasahiroBoege, FritzMishra, Pawan KumarBoeglin, AlexMishra, Rupesh K.Boekema, BoukeMisra, Biswapriya BiswavasBoerma, MarjanMitani-Ueno, NamikiBoesze-Battaglia, KathleenMitchell, Cassie S.Bogusławska, JoannaMitchell, JohnBøgwald, JarlMitoma, HirokiBoichuk, SergeiMitrakas, LamprosBoitel-Conti, MichèleMitrofanis, JohnBojarová, PavlaMitroulis, IoannisBojková, BiankaMitsuya, ShiroBokareva, Olga S.Miura, KazukiBombard, SophieMiura, NaokiBommagani, ShbanbabuMiura, SayakaBommakanti, GayathriMiyagishima, Shin-yaBonini, Sara AnnaMiyake, MakitoBonnelye, EdithMiyamoto, HirotakaBonnet, Pierre-AntoineMiyamoto, KojiBonnet, SébastienMiyamoto, ToshinobuBonni, ShirinMiyata, HiroshiBonomini, FrancescaMiyata, YasuyoshiBonora, ElenaMiyazaki, TeruoBontempo, PaolaMizuno, Cassia S.Boomer, Jonathan S.Mizuno, KentaroBora-Singhal, NamrataMizuno, MasashiBorcan, FlorinMizuno, TooruBorchman, DouglasMizzotti, ChiaraBordin, LucianaMladěnka, PřemyslBordonaba, Jordi GineMlejnek, PetrBorer, Katarina T.Mlera, LuwanikaBorghaei, Ruth C.Mlocicki, DanielBorghini, AndreaMo, Yin-YuanBorin, Thaiz FerrazMoccia, FrancescoBorkowski, LeszekMock, BeverlyBoros, LaszloMock, Hans-PeterBorovac, Josip A.Modepalli, VengamanaiduBorsani, ElisaModest, Dominik PaulBortolotti, CarloModhiran, NaphakBortoluzzi, StefaniaMody, HardikBoscaiu, MonicaMoeller, LarsBosch, MauriceMoertl, SimoneBoschetti, FedericaMogi, MasakiBoschi-Muller, SandrineMohamed, IslamBoschin, GiovannaMohamed, Salah A.Boscia, FrancescaMohammadi, FakhrossadaBose, DebojitMohammadiarani, HosseinBose, SayantanMohammed-Geba, KhaledBosland, Maarten C.Mohan, Ganesh Babu MalliBosnjak, BerislavMohanta, TapanBossard, PascaleMohd, Farooq ShaikhBosse, Jens B.Möhlendick, BirteBossi, FleurMohsin Tanveer, MohsinBossi, GianlucaMoir, Robert D.Botelho, Monica C.Moisa, SoniaBotturi, AndreaMolina, Cristina E.Bouché, MarinaMolinier, JeanBoudhraa, ZiedMolino, PaulBourgault, SteveMollapour, MehdiBousbaa, HassanMoller, IanBoutin, JeanMollerup, SteenBoutin, Jean A.Monaghan, JamesBouvier, BenjaminMonastyrskaya, KatiaBowen, JamesMoncayo, GeraldBowen, TimMoncayo, RoyBowerman, MelissaMoncho Jorda, ArturoBowling, HeatherMonetta, TullioBoyer-Guittaut, MichaëlMonferran, SylvieBoyle, Aimee L.Mong, Mei-ChinBozzi, FabioMongiat, MaurizioBraakhuis, AndreaMoni, LisaBrabec, ViktorMoniuszko-Szajwaj, BarbaraBracci, MassimoMonji, AkiraBracco, EnricoMontag, JudithBrack, AndréMontalvo-Rodríguez, RafaelBradbury, AllisonMonteiro, AlvaroBradbury, RichardMonteiro, SusanaBrady-Kalnay, Susann M.Montes Resano, MartaBraig, SimoneMontesarchio, DanielaBrait, MarianaMonticone, SilviaBranca, Rosa TamaraMooers, BlaineBranchini, AlessioMoon, IlsooBrandão, Rita DiasMoore, PeterBrandenburg, KlausMoore, StuartBrandli, AliceMorales Suárez-Varela, María M.Brands, Michael W.Morales, JulioBrandt, RolandMorales-Tirado, VanessaBratlie, Kaitlin M.Moran, OscarBraun, LaurenceMorandi, LucaBravo, JeronimoMoras, DinoBrecht, Jeffrey K.Morbidelli, LuciaBrehmer, AxelMoreau, KevinBreitbart, HaimMoreau, MarcBreitenfeld Granadeiro, LuizaMoreira, PaulaBrem, JurgenMorello, SilvanaBren, UrbanMoreno, Diego A.Brender, JeffreyMoreno, J. J.Brenner, Annette K.Moreno-Manzano, VictoriaBrenner, RobertMoretti, MartaBrestic, MarianMorfini, GerardoBrestoff, Jonathan R.Morgan, AngelaBreuzard, GillesMorgese, Maria GraziaBrevern, AlexandreMorgutti, SilviaBrew-Appiah, RhodaMori, IzumiBrewer, MeganMori, KazuhiroBrigelius-Flohe, ReginaMori, MattiaBrignole, ChiaraMoricoli, DiegoBril, FernandoMorigi, RitaBrinchmann, Monica F.Moriguchi, TakayaBrink, PeterMorikawa, ToshioBritto, Dev T.Morimoto, MasanoriBriza, PeterMorimoto, NobuyukiBrizzi, Maria FeliceMorimoto, TatsuyaBroderick, PatriciaMorimura, ShigeruBroeckling, CoreyMoriones, EnriqueBroer, StefanMorita, ManabuBrogi, SimoneMorita, MitsuhiroBronisz, AgnieszkaMorita, NaokiBross, PeterMorita, ShigetoBrown, Euan R.Moriya, TakahiroBrown, GeoffreyMorkunas, IwonaBrown, HannahMorozov, Alexander N.Brown, Keith W.Morris, BrianBrown, Robert J.Morris, Brian J.Brozyna, AnnaMorrison, MelanieBruce, Alexander W.Morrison, PeterBrullo, ChiaraMortara, LorenzoBrumos, JavierMortiboys, HeatherBruna, AlejandraMorton, PaulBruneel, ArnaudMortusewicz, OliverBrunelli, ElviraMoschovi, MariaBrunet, FrédéricMostofa, Agm G. M.Brunnström, HansMota, João FelipeBruns, HeikoMota, ManuelBruschi, FabrizioMotorin, Yuri A.Bruserud, ØysteinMottes, MonicaBrusslan, JudyMou, HaiweiBryant, Andrew J.Moulder, RobertBryszewska, MariaMoulin, LionelBrzóska, EdytaMourad, RaphaelBrzozowska, EwaMouriño, AntonioBrzozowski, ThomasMousa, AyaBubb, Kristen J.Mouzeyar, SaïdBubici, GiovanniMovahedi, AliBubulya, Paula A.Mrakovcic, MariaBucciantini, MonicaMrazek, JanBuccigrossi, VittoriaMrówczyński, RadosławBuchheim, Judith-IrinaMucignat-Caretta, CarlaBuchoux, SébastienMueller, GeoffreyBucki, RobertMuench, GeraldBuckley, M.Mühle, ChristianeBucolo, ClaudioMujtaba, ShirazBudinsky, Robert A.Mukai, ShoichiroBudnyak, TetyanaMukaida, NaofumiBudryn, GrażynaMukherjee, Sudipbuechler, christaMukohda, MasashiBueno, MartaMulas, MaurizioBuettner, AndreasMulero, VictorianoBugla-Płoskońska, GabrielaMüller, JensBugliani, MarcoMuller, MarcBüki, AndrasMuller, Patricia A. J.Bulli, PeterMüller, RüdigerBullon, PedroMullin, James MBunaciu, Rodica P.Mullins, Robert F.Bungartz, GerdMulloy, BarbaraBunt, JensMulo, PaulaBuravkova, LudmilaMummadisetti, ManjulaBurchell, JoyMunaron, LucaBurdo, Tricia H.Munday, MichaelBurger, MichaelMuñoz Caffarel, MaríaBurger, RenateMuñoz, ManuelBurghardt, KyleMuñoz-Torrero, DiegoBürglin, Thomas R.Münscher, AdrianBurgstaller, JoergMünsterberg, AndreaBurke, DermotMunteanu, Florentina-DanielaBurke, RichardMupo, AnnalisaBurlingham, William J.Muraca, MaurizioBurns, Jorge S.Murakami, YasufumiBurtea, CarmenMuraki, KatsuhikoBurtey, StephaneMurata, JunBurtey, StéphaneMurata, TakayukiBusby, ChrisMurcha, MonikaBusch, HaukeMurdaca, GiuseppeBusinaro, RitaMurdoch, ColinBussolati, OvidioMurgia, MartaBusson, PierreMurotomi, KazutoshiButi, MatteoMurovska, ModraButterfield, Russell J.Muroya, SusumuBüttner, A.Murphy, CormacBystrowska, BeataMurphy, EainByun, JonghoeMurray Stewart, TracyBywater, Robert P.Muruganandan, ShanmugamCaarls, LotteMusch, MarkCabrele, ChiaraMusgrave, IanCabrera, AdoracionMustacchi, GiorgioCabrera-Fuentes, Hector AlejandroMustafa, GhazalaCaccamo, DanielaMusumeci, GiuseppeCaccetta, RimaMus-Veteau, IsabelleCacciotto, CarlaMuszyński, ArturCacciottolo, MafaldaMuthuramu, IlayarajaCadamuro, MassimilianoMuti, PaolaCaeiro, MariaMutsaers, Steven E.Caetano, NídiaMutti, LucianoCaffarelli, CarloMyers, StephenCahoon, Aubrey BruceMyllykallio, HannuCai, LuMysiorek, CarolineCaiazzo, ElisabettaMyśliwiec, HannaCairrão, ElisaMystkowska, JoannaCaja, LaiaN. Setzer, WilliamCalarco, AnnaNa, DokyunCalcutt, MichaelNadia, BazihizinaCaldo, Kristian MarkNadia, RucciCalhelha, Ricardo CostaNagai, KouheiCalì, TitoNagai, NoriakiCalkin, AnnaNagai, TakayukiCall, MindyNagaraj, ChandranCalle-Pascual, Alfonso L.Nagarajan, SureshbabuCallery, Patrick S.Nagata, NaotoCalloe, KirstineNagata, ShinjiCalvano, Cosima DamianaNagata, ToshiCalvo-Lobo, CésarNagatani, NaokiCâmara, José SousaNagpal, RavinderCamejo, GermánNagy, EndreCamire, MaryNagy, IstvanCammarata, KirkNagy, PeterCampanini, BarbaraNah, Jae-WoonCampbell, CoreyNahashon, SamuelCampbell, MichaelNaik, SurabhiCampbell, MorayNair, Jijeesh RaviCampione, MarinaNair, ShinyCampos, EricNajimi, MustaphaCampuzano, OscarNajlah, MohammadCancemi, PatriziaNakabayashi, KazumiCandido Carvalho, KatiaNakagawa, AtsushiCândido, ElizabeteNakagawa, TakuroCanela, NúriaNakagawa, TsuyoshiCanetta, ElisabettaNakahama, Ken-ichiCangkrama, MichaelNakajima, MasatoshiCanini, AntonellaNakamaru-Ogiso, EikoCannavo, AlessandroNakamura, HitomiCannone, ValentinaNakamura, YoshikazuCanonico, MarianneNakanishi, AkiraCansby, EmmelieNakano, HiroyasuCantalapiedra, Carlos P.Nakano, ToshiakiCantini, FrancescaNakao, ReikoCao, RenzhiNakashima, KazuoCao, TongyuNakashima, SouichiCao, WeiguoNakashiro, Koh-IchiCao, ZheNakatsuka, A.Capanoglu, EsraNakatsura, TetsuyaCaparros-Martin, JoséNakayama, YujiCapasso, RaffaeleNakazawa, YasumotoCapel, CarmenNalam, VamsiCapel, FredericNallasamy, PalanisamyCapel, FrédéricNalluri, JosephCapitanio, NazzarenoNam, Jin-WuCappello, PaolaNanavati, CharviCapranico, GiovanniNanba, KazutakaCaprini, MarcoNandha Premnath, PadmavathyCaputo, Gregory A.Nandi, SaikatCaputo, RobertoNanjundan, MeeraCaraglia, MicheleNanni, LorisCarapito, RaphaelNano, AdelaCarballo, IriaNaoumkina, MarinaCarballo, SebastianNapoli, EleonoraCarbone, CarmineNapolitano, MariasantaCarbonell, TeresaNaponelli, ValeriaCarboni, LuciaNarasimhan, MadhusudhananCardenas Zuniga, RobertoNarayanan, AjitCardia, Maria CristinaNarayanan, AnandCardin, ChristineNardelli, CarmelaCardoso, Fernanda CaldasNardi, SerenellaCardoso, HéliaNarkar, Vihang A.Cardoso, SusanaNaruishi, KojiCarella, PhilipNarute, PurushottamCaretta, AntonioNarváez, ManuelCariati, FedericaNaryzhny, StanislavCarillo, PetroniaNashima, KenjiCarlberg, CarstenNass, NorbertCarletti, PaoloNassuth, AnnetteCarlisi, DanielaNastri, FlaviaCarlsbecker, AnnelieNataf, SergeCarmela, NardelliNatarajan, MohanCarmosino, MonicaNatarajan, Sathish KumarCarobbio, StefaniaNatera-De Benito, DanielCarol, PierreNatile, GiovanniCarosio, FedericoNault, RanceCarotenuto, MarcoNaumann, Todd A.Carpaneto, ArmandoNaumenko, Vladimir S.Carpene, ChristianNavab, MohamadCarpenter, G. H.Navarro, EstanisCarpenter, RichardNavarro, ManuelCarradori, SimoneNavazio, LorellaCarraher, CharlesNaveira, HoracioCarranca, CorinaNaviglio, SilvioCarrasco, PedroNawaz, MuhammadCarregal, SusanaNayak, TapanCarrer, AlessandroNaylor, KariCarreras, JoaquimNaz, AliCarrie, ChrisNazar, Ross N.Carrie, GrueterNazarov, AlexeyCarrillo-Sepulveda, Maria AliciaNazimek, KatarzynaCarroll, PatrickNduhirabandi, FredericCarroll, VeronicaNeale, ElizabethCarson, RayNedialkov, Paraskev TodorovCarta, FabrizioNeeland, MelanieCarta, GianfrancaNees, MatthiasCartagena-Rivera, AlexanderNeesen, JuergenCarter, WayneNegishi, TakayukiCartland, SianNegroiu, GabrielaCaruso, CalogeroNeira, José L.Carvajal Moreno, FátimaNelson, Amanda M.Carvajal, MicaelaNelson, AndrewCarvalho Pimenta, DanielNelson, Fred R. T.Carvalho, KatherineNemerow, GlenCarvalho, LuísaNémeth, BalázsCasadei, RaffaellaNepal, MadhavCasal, Margret L.Neri, SimonaCasal, SusanaNesmelov, Yuri E.Casals, NúriaNesmelova, IrinaCasamassimi, AmeliaNeuhaus, JochenCasaretto, Jose A.Neumann, CarolaCasas, Ana M.Neureiter, DanielCasati, LaviniaNeutelings, GodfreyCascio, SandraNewburg, DavidCascione, MariafrancescaNexø, EbbaCaser, MatteoNg, AndyCasimiro, SandraNg, Li FangCasini, GiovanniNg, RayCassina, ValeriaNgamcherdtrakul, WorapolCastagna, MichelaNguyen, CuongCastaldi, AlessandraNguyen, Khue VuCastanera, RaúlNi, HongminCastanheira, Elisabete M. S.Nicholls, Stephen J.Castegna, AlessandraNicholson, John W.Castellano, OrlandoNicolay, NilsCastellone, Maria DomenicaNicoletti, FerdinandoCastilho, AlexandraNicolini, AndreaCastorina, AlessandroNicosia, AldoCastresana, JavierNieberler, MarkusCastro, Antonio JesusNiederman, Robert A.Castro, RitaNiehaus, TomCastrucci, Ana Maria De LauroNielsen, Morten SchakCattaneo, FabioNielsen, Vance GirardCatucci, LuciaNiemeyer, Emily D.Cauli, OmarNiemirowicz-Laskowska, KatarzynaCava, ClaudiaNieto, PedroCavalcanti, João Henrique F.Nieto, Teresa PérezCavallo, CarolaNieves-Cordones, ManuelCavani, LucianoNiewiadomska, EwaCebova, MartinaNifli, Artemissia-PhoebeCeccarelli, SaraNigris, Filomena DeCeccherini, Maria TeresaNijhof, IngerCecchetti, SerenaNikodemova, MariaCecchinato, ValentinaNikolaev, ViacheslavCécile, OuryNikolakaki, EleniCecilia, Juan AntonioNikolakopoulou, Angeliki M.Cederbaum, ArthurNiland, StephanCefalas, A. C.Nilsen, Erik T.Cehofski, LasseNima, Zeid A.Celiński, KonradNirichan, Sanoj RejinoldCelli, AnnaNishi, MayumiČėnas, NarimantasNishida, NaokiCeolotto, GiulioNishigaki, TakuyaCeranowicz, PiotrNishiguchi, MasamichiCerchiara, TeresaNishihara, TatsujiCeredig, RhodriNishimura, KaneyasuČerný, JiříNishizuka, MakotoČerný, MartinNisiotou, AspasiaČeřovský, VáclavNisnevitch, MarinaCerra, BrunoNissinen, RiittaCerutis, RoselynNistala, RaviCerutti, FrancoNitiss, JohnCervelló, IreneNitulescu, George MihaiCervera, AmeliaNiu, JingwenCésar, Ibarra-AlvaradoNizhnikov, Anton A.Cestmir, AltanerNobili, StefaniaCha, ChaenyungNoce, AnnalisaCha, Hee-JaeNocentini, GiuseppeChabé, MagaliNociari, MarceloChae, HeeyoungNoghero, AlessioChaerle, LauryNoguchi, Constance TomChaganty, BharatNoguchi, MasayukiChai, YunrongNoguchi, TakuyaChakrabarti, KausikNohe, AnjaChakraborty, ArupNonami, AtsushiChakraborty, RajaNonnekens, JulieChakraborty, SayanNonnemacher, Michael R.Chakravorty, DavidNoordermeer, DaanChalah, MoussaNorata, Danilo GiuseppeChalah, Moussa AntoineNoren Hooten, NicoleChalivendra, Subbaiah C.Norihiko, NaritaChalkia, DimitraNorman, Kenneth R.Challa, DilipNorman, TrevorChamcheu, Jean ChristopherNorris, ErinChamonine, MikhailNorton, MichaelChampion, AntonyNosetti, LuanaChamuleau, Robert A. F. M.Nosoudi, NasimChan, Ben C. L.Notas, GeorgeChan, Chi HoNovellino, EttoreChan, Chun WaiNovello, VittorinoChan, Hon FaiNovick, DanielaChan, Michael WYNovikov, AlexanderChandaka, Giri KumarNovikova, PolinaChang, Chia-cheNovio, FernandoChang, Chien-ChungNovío, SilviaChang, Chuang-RungNowak, AdrianaChang, DavidNowak, Felicia V.Chang, Fu-KueiNowak, RenataChang, Hao-XunNowak-Sliwinska, PatrycjaChang, Hsueh-WeiNowicka, GrażynaChang, Hui-huaNowicki, JanuszChang, Hung-YuNowicki, MarcinChang, Ing-FengNowotarski, ShannonChang, Kee-LungNowotny, NorbertChang, Ko-TungNozaki, MasamiChang, Kuan Y.Ntalli, Nikoletta G.Chang, Long-SenNtatsi, GeorgiaChang, Shun-FuNtie-Kang, FideleChang, Te-ShengNudler, EvgenyChang, Wei-JenNumakawa, TadahiroChang, Wen-ChiNutt, Stephen L.Chang, Wen-WeiNuytemans, KarenChanson, MarcNyberg, FredChao, Louis KuopingNylander, KarinChapman, EliNylandsted, JesperChapoval, Svetlana P.Nyström, KristinaCharbonneau, MichelO’Brien, EdwardChardon, FabienO’Carroll, Simon J.Charlton, ClivelOak, NinadChase, ChristineOakhill, Jonathan S.Chass, GregoryObana, AkiraChater, CasparObata, HideakiChatfield, DavidOberg, Kerby C.Chatterjee, NimratObermeier, BirgitChaudhuri, NaziaObermüller, NicholasChauhan, ArunObiang-Obounou, Brice WilfriedChaurand, PierreOchi, ShinichiroChaves, ElenaOchieng, JosiahChaves, RaquelOchoa-Repáraz, JavierChaves-Martinez, Felipe JavierO'Connell, CatherineChavez, FermanO'Connell, RichardChe, C. -T.O'Connor, ChristineCheca, AntonioOćwieja, MagdalenaChekanova, Julia A.Oda, YukariChelbi, Sonia T.O'Doherty, GeorgeChen, ChiachungOeckinghaus, AndreaChen, Chih-HaoOfford, JamesChen, Chun-JungOgasawara, ToruChen, Chun-LiangOgawa, AkikoChen, GuangpingOgbourne, StevenChen, Guan-YuOgden, Stacey K.Chen, GuoxunOgi, KazuhiroChen, Gwo-ShingOgino, ShujiChen, HongweiOgórek, RafałChen, Hui-WenOgunjirin, AdebowaleChen, Jen-TsungOh, Han BinChen, JiangangOh, JisunChen, JianzhongOh, JonghyunChen, Ko FanOh, Man-hoChen, Kuan-FuOh, YohanChen, LinOh, Yoon SinChen, MingO'Hagan, Heather M.Chen, Min-HueyOhashi, MiwaChen, Po-chiaOhe, KenjiChen, Rong-JaneOhkuri, TakayukiChen, RuiOhlsson, BodilChen, ShuyangOhmiya, AkemiChen, SuzieOhnishi, MasatoshiChen, TianbaoOhno, ShoChen, Tzong-YuehOhshimo, ShinichiroChen, WeiqinOhtomo, RyoChen, Wei-ShengOhya, SusumuChen, Wei-YuOikawa, AkiraChen, WenchunOing, ChristophChen, WenwenOjala, JohannaChen, XiaozhuoOka, ShuntaroChen, Ya-LeiOkada, KazunoriChen, YanaOkada, MuneyoshiChen, Yann-JangOkada, TaketoChen, YiliangOkada, TomoyoChen, Yi-NingOkada, YasunobuChen, Yi-WenOkajima, HideakiChen, Yu-ChihOkamoto, MasamiChen, Yung-ChihOkamoto, TakayukiChen, Yung-HsiangOkazaki, ToshiroChen, Yun-WenOkazawa, AtsushiChen, ZheOkleštková, JanaChen, ZhihangOkui, TatsuoCheng, DeOkumura, NobuakiCheng, HailiOkunishi, KatsuhideCheng, Hung-ChiOlaru, Octavian TudorelCheng, Juei-tangOlejnik, AnnaCheng, Jya-WeiOleskin, Alexander V.Cheng, Kuang-HungOliveira, JorgeCheng, PeiOliveira, Paula Alexandra Martins DeCheng, ShouyunOliveira, RaquelCheng, XiaomingOliver, BrianCheng, XiaoyanOliver, PaulaCheon, Choong-illOlmos, DaniaCheon, Yong-PilOlson, Michael E.Cheong, Eun JuOlsson, SimonCheong, Mi SunOltean, MihaiCheong, Yong HwaOltra, ElisaCheong, Young HunOmais, SaadChereddy, Kiran KumarOmar, AhmadCherry, JonathanOmar, HanyCheung, MartinOmar, Syed HarisChhabra, GaganOmidvar, VahidChi, GeraldOMORI, YasufumiChiabrando, Gustavo AlbertoOniszczuk, AnnaChiam, KarenOnorati, Maria CristinaChiang, Hsiu-MeiOnuta, Marie-ChristineChiang, Yi-TingOnyenwoke, Rob UChiao, Ying AnnOon, Hazel H.Chiappinelli, KatherineOono, YoukoChiaraluce, RobertaOosterwijk, EgbertChiaravalli, Anna MariaOpalińska, MagdalenaChiarelli, LaurentOpitz, JörgChichger, HavoviOracz, JoannaChiefari, EusebioOrchard, SandraChiellini, GraziaOrenstein, YaronChien, Hung-YuOrio, LauraChien, Yin-HsiuOrlandi, AugustoChien, Yi-wenOrlov, SergeiChiessi, EsterOrosa, BeatrizChifiriuc, Mariana CarmenOrren, David K.Chilian, WilliamOrsavová, JanaChin, Young-WonOrsolic, NadaChinnappan, MahendranOrtega, Eugenio VelascoChio, ChristineOrtega, Maria TeresaChiocca, SusannaOrtega, NatividadChiodini, IacopoOrtolano, SaidaChiou, Chun-TangOrton, Thomas J.Chiricozzi, AndreaOry, StéphaneChitraju, ChandramohanOrzechowski, ArkadiuszChiu, ChunOsanai, TomohiroChiu, Ing-MingOshima, YusukeChiu, JoannaOshiumi, HiroyukiChiu, Sheng-ChunOsses, NelsonChiu, Wen-TaiOstaszewski, RyszardChiu, Ya-HuiOster, HenrikChiurchiù, ValerioOstersetzer-Biran, OrenChladek, GrzegorzOstuni, MarianoChlichlia, KaterinaOszust, KarolinaChmurzynska, AgataOtani, KeisukeCho, HongsikOthumpangat, SreekumarCho, HyosunOtsuka, YuzuruCho, HyunahOtsuki, TakemiCho, Je-YoelOtulak-Kozieł, KatarzynaCho, Yoon HeeOtyepka, MichalChobot, VladimirOu, Keng-LiangChoi, BogyuOwczarczyk-Saczonek, AgnieszkaChoi, Kyung-ChulOzaita Mintegui, AndrésCholkar, KishoreOzaki, ToshinoriChong, ParksonOzeki, YasuhiroChorley, BrianOzen, Mehmet OzgunChou, Cheng-YangPabbidi, Reddy MChou, Feng-ChengPacak, AndrzejChou, Lan-SzuPacal, LukasChoudhary, Kumari SonalPacchierotti, FrancescaChoudhury, MalayPachapur, VinayakChoudry, Haroon A.Pachter, Joel S.Chowdhury, AnandaPaci, MaurizioChoy, Francis Y. M.Pacifici, MaurizioChrist, BrunoPacini, NicolaChrist, TorstenPacios, Luis F.Christensen, ShawnPackiam-Dayalan, AntonyChristians, UwePadula, MatthewChristodoulou, Maria-IoannaPaeschke, NadineChristoffers, MichaelPaeshuyse, JanChruszcz, MaksymilianPagán, IsraelChu, Ching-LiangPagani, StefaniaChu, JiaxiangPagano, FrancescaChu, Pei-MingPagano, LucaChu, Shi-JyePagano, StefanoChu, XiangpingPage, MelissaChua, Melvin L. K.Paggetti, JérômeChuang, Hung-YiPagliai, Fernando A.Chui, Kwok TaiPagliarani, ChiaraChun, Kyung-SooPain, BertrandChung, Brian K.Paja̧ķ, PaulinaChung, Eun JooPakdel, FarzadChung, Jay H.Pal Choudhuri, ShreoshiChung, Yong-AnPal, KasturiChwalibog, AndréPál, MagdaChwastek, GrzegorzPalamà, IlariaChyau, Charng-CherngPalandri, FrancescaCiaccio, MarcelloPalaniappan, BalasubramanianCiancetta, AntonellaPalanisamy, ArulselvanCianci, AntonioPałasz, ArturCiarimboli, GiulianoPalese, PeterCicciù, MarcoPalioura, SotiriaCiccone, Marco MatteoPalma Sircili, MarceloCicero, Arrigo Francesco GiuseppePalmblad, MagnusCichero, ElenaPalmeira, Carlos ManuelCichorek, MiroslawaPalmieri, CamilloCiciliot, StefanoPalmieri, OrazioCieniewski-Bernard, CarolinePalmirotta, RaffaeleCieplik, FabianPalomba, LetiziaCiereszko, IwonaPalomo Ríos, ElenaCifone, Maria GraziaPalumbo, CarlaCigana, CristinaPalumbo, PaolaCignarella, AndreaPalumbo, Vincenzo DavideCignetti, AlessandroPaluszczak, JarosławCimmino, FloraPan, HaihuaCimmino, GiovanniPan, Hung-ChuanCîmpean, AnişoaraPan, MiaoCioffi, MarceloPan, PanCione, ErikaPan, Ping-YingCiregia, FedericaPan, Szu-HuaCiriaco, FulvioPan, Y. J.Ciribilli, YariPan, YangangCirilli, MarcoPanagiotidis, MihalisCiriza, JesusPanaro, Maria AntoniettaCisowsky, JaroslawPanatala, RadhakrishnanCitek, JindrichPancsa, RitaCitro, ValentinaPanda, Amaresh C.Cividini, FedericoPandit, HarshulCizkova, DasaPandolfi, AssuntaClaesson-Welsh, LenaPandolfini, TizianaClanchy, FelixPanfoli, IsabellaClapp, Lucie H.Pang, BoClarke, AnthonyPang, Myung-GeolClarke, GerardPang, YoonsooClausen, JohannesPani, BibhusitaClavé, PerePanikkanvalappil, Sajanlal R.Clayton, AndrewPantazaki, Anastasia A.Clelland, CatherinePanwalkar, PoojaClement, SophiePanzarini, ElisaCliment, MontserratPanzella, LuciaCoan, P. M.Paoletti, Anna MariaCobellis, GildaPaolillo, MayraCochis, AndreaPaolo Busardó, FrancescoCognasse, FabricePapa, AlfredoCohen, Ari J.Papaccio, GianpaoloCohen, DavidPapadimitriou, KonstantinosCohen, MichaelPapadodima, OlgaCohen-Salmon, MartinePapamatheakis, Joseph (Sifis)Coiffard, Laurence J. M.Papantonopoulos, GeorgeColdea, TeodoraPapazafiropoulou, AthanasiaColecchia, DavidPapenbrock, JuttaColella, MatildePapet, IsabelleColey, Helen M.Papini, AlessioCollavin, LicioPapp, AndrásCollier, JasonPappas, Maria L.Collin, MatthewParadowska-Gorycka, AgnieszkaCollins, ColmParashar, DeepakCollins, Jennifer J. P.Parayath, Neha N.Colombo, EmanuelaPardali, EvangeliaColombo, MonicaPardi, NorbertColonna, GiovanniPardo, Jose M.Colotti, GianniParfrey, HelenColquhoun, Thomas A.Parikesit, Arli AdityaColuccia, MauroParine, Narasimha ReddyColussi, GianLucaParisi, LudovicaCombarnous, YvesParisini, EmilioComi, CristoforoPark, Byung-WookCominelli, EleonoraPark, Dong SunCommisso, MauroPark, EnochCondorelli, Daniele FilippoPark, Hyun-WooConstantin, LucianPark, JiyoungConti, BicePark, Joo-CheolConti, HeatherPark, JoshuaConti, PioPark, JuhyunContreras, RúbenPark, JunsooCook, LeahPark, Ky YoungCook-Mills, JoanPark, KyeongsoonCooper, Arthur J. L.Park, Kyoung ChanCooper, RobinPark, MaiyonCooper-Knock, JohnathanPark, Sang WonCopolovici, Dana MariaPark, Sung HaCoppedè, FabioPark, Sung-GyooCoquet, LaurentPark, Tae HwanCorazzari, MarcoPark, WansuCorbet, CyrilPark, YoonseongCordaro, MarikaParker, DaneCordelier, SylvainParolini, CinziaCorfield, Anthony P.Parrella, EdoardoCorke, HaroldParrizas, MarcelinaCormerais, YannParsons, JasonCornetti, LucaPartanen, JukkaCornillet, MartinParthasarathy, SrinivasCorrado, ChiaraPartsch, HugoCorre, IsabelleParveen, IffatCorrigan, FrancesPascual, AlbertoCortés Castell, ErnestoPasini, LuigiCorthay, AlexandrePassamonti, SabinaCorvalan, AlejandroPassarinha, Luís António PaulinoCos, PaulPassini, ElisaCosco, DonatoPasternak, TarasCosentino, CarloPasternak, Taras P.Cosme, MarcoPastor, FernandoCosta, MarinaPastores, Gregory M.Costa, Vera MarisaPaszek, PawelCosta-Hurtado, MarPatankar, Manish S.Costantini, SusanPatel, Daxesh P.Costantino, ValeriaPatel, DevenCotella, DiegoPatel, HardikkumarCottrell, JeremyPatel, Yashomati M.Coumoul, XavierPaterson, David J.Courtois, GillesPathakoti, KavithaCoustham, VincentPathania, RajneeshCoutinho, João PauloPatil, AbhinandanCoutinho, Maria FranciscaPatil, Naeem K.Covello, GiuseppinaPatrick, RalphCovino, RobertoPatruno, MarcoCowardin, CarriePatterson, Eric L.Cowell, IanPatterson, JenniferCowell, RitaPaul, AnanyaCowin, AllisonPaul, Narayan ChandraCox, Linda S.Paulin, RoxaneCrawford, Bryan D.Paulo, AntónioCrawford, LindseyPauwels, LaurensCrawford, LisaPavic, ValentinaCrawley, Angela M.Pavlik, EdwardCreixell, Teresa AdellPavlov, DanailCrescenzi, MarcoPawelkowicz, MagdalenaCrespan, EmmanuelePawlak, DariuszCrespo, Mariano SánchezPawlak, MichalCridge, AndrewPawłowski, Tomasz A.Crisci, AlessandroPayan-Carreira, RitaCrisponi, GuidoPayne , AnnetteCristofori-Armstrong, BenPazdro, RobertCrnkovic, AnaPazzagli, LuigiaCroce, Anna CletaPearen, MichaelCrompton, TessaPease, James B.Crosatti, CristinaPébay, AliceCroset, MartinePečenková, TamaraCrucho, Carina Isabel CorreiaPechanova, OlgaCruz-Topete, DianaPécheur, Eve-IsabelleCsányi, GáborPeck, AmmonCsiszar, AgnesPedatella, SilvanaCsiszár, AgnesPeddi, PrakashCsősz, ÉvaPedeux, RémyCubero, JavierPedone, ElisaCucchiarini, MagaliPedrazzini, EmanuelaCuello, FriederikePedroza, MesiasCui, FangdaPeet, DanielCui, HaitaoPeffley, Dennis M. Cukrov, DubravkaPegg, Anthony E.Cukrowska, BoženaPegler, Joseph L.Culi, JoaquimPego Reigosa, Jose MariaCullis, ChristopherPeguero-Pina, JoséCupellini, LorenzoPeguero-Pina, José JavierCuppoletti, JohnPeigneur, SteveCurtale, GraziellaPeiró, SandraCusanelli, EmilioPeitzsch, ClaudiaCutuli, DeboraPejoski, DavidCvikl, Doz. BarbaraPel, JoelCyganowski, PiotrPelagalli, AlessandraCynis, HolgerPelin, MarcoCzaja, KrzysztofPellegrini, Gretel G.Czarnecka, BeataPellice, SergioCzeglédi, LeventePellis, AlessandroCzerwinska, MonikaPelosi, PaoloCzikora, IstvanPemp, BertholdCzubryt, Michael P.Peña, Amado SalvadorCzyż, JarosławPeña, Maria Marjorette O.Czyż, KatarzynaPendyala, GuruduttD’Amico, Agata GrazianaPenfornis, PatriceD’Angelo, PaolaPeng, ChenDa Cruz, SandrinePeng, Sheng-BinDaberkow, DaberkowPeng, TengDacheux, Jean-LouisPeng, Wen-HuangDachineni, RakeshPeng, XinxiaD'Acunto, Cosimo WalterPenke, BotondDaczewska, MałgorzataPenna, FabioDada, NsaPennock, NathanDagda, Ruben K.Pentzold, StefanD'Agostino, NunzioPenuela, SilviaDai, ShaojunPenzo, MariannaDai, Yang D.Perche, FédéricoDaimiel, LidiaPerego, PaolaDakhlallah, DuaaPereira, BrunoDal Ben, MatteoPereira, Carlos DiasDal Col, JessicaPereira, Cláudia Maria F.Dal Piaz, FabrizioPereira, Olívia R.Dalbo, VincentPerepelyuk, MarynaDalin, Martin G.Perera, OmaththageDallacosta, LorenzaPérez Alvarez, Maria JoséDallinger, ReinhardPerez Pascual, DavidDamia, GiovannaPerez, Juan J.D'Amora, UgoPérez, SoledadDandekar, ThomasPerez-Clemente, Rosa MariaDane, FennyPérez-García, SeleneDaniels, MatthewPérez-Montaño, FranciscoDanila, Daniel C.Pérez-Pérez, José ManuelDanilenko, MichaelPerez-Stable, CarlosDanilov, AlexanderPeri, FrancescoDansette, Patrick M.Perinelli, Diego RomanoDante, SilviaPeriyasamy, PalsamyDaras, GerasimosPerl, AndrasDarbani, BehroozPermyakov, EugeneD'Argenio, ValeriaPerni, MicheleDarias, MaríaPernisová, MarkétaDarkó, ÉvaPero, RaffaelaDarling, April L.Perrella, GiorgioDarling-Reed, Selina F.Perricone, CarloDarvell, Brian W.Perrine-Walker, FrancineDas, AditiPerry, RachelDas, AninditaPersaud, KrishnaDas, LalitenduPerteghella, SaraDas, ViswanathPerticone, FrancescoDasgupta, KasturiPerucka, IrenaDasgupta, SantanuPervouchine, DmitriDash, BirajaPešić, MilicaDash, RanjeetPessi, GabriellaDatta, PoulamiPestov, DimitriDatta, RupsaPeter, FranciscDaugrois, Jean-HeinrichPETER, Martin G.Dave, SandeepPeters, EvaDavey, Matthew P.Peters, VerenaDavid Greg, RileyPetersen, BjörnDavid, AlexandrePetersen, Kristina S.Davidson, CristinPeterson, BrandonDavinelli, SergioPeterson, JuliaDavis, AdamPeterson, LarrynDavis, CatherinePetit, Patrice X.Davletov, BazbekPetra I., LorenzoDavoli, RobertaPetras, DanielDawood, Mahmoud A. O.Petrella, AntonelloDawson, GlynPetri Jr., William A.Day, DavidPetroni, KatiaDe Almeida, AndreiaPetrussa, ElisaDe Bellis, LuigiPetrzik, KarelDe Berardis, DomenicoPetta, IoannaDe Biase, DanielaPflugfelder, Stephen C.De Bock, MarijkePhang, MelindaDe Carlo, FlaviaPhatak, PornimaDe Chiara, LetiziaPhesse, TobyDe Craene, Johan-OwenPhilipp, StephanDe Deurwaerdère, PhilippePhilips, NeenaDe Diego, NuriaPhilpott, MichaelDe Feo, VincenzoPich, ChristineDe Filippis, VincenzoPicimbon, Jean-FrançoisDe Francesco, ErnestinaPicketts, David J.De Franciscis, PasqualePickrell, AliciaDe Gonzalo-Calvo, DavidPidatala, Venkata RamanaDe Graaf, Barend H. J.Pidgeon, Graham P.De Guzman Strong, CristinaPiedade, Ana PaulaDe La Chapelle, AlbertPiedras, PedroDe La Fuente, HortensiaPiemontese, LucaDe La Fuente-Nunez, CesarPieraccini, StefanoDe La Grange, Philippe BrunetPierangeli, AlessandraDe La Herrán, RobertoPierre, SandrineDe La Luz Garcia-Hernandez, MariaPierrefite-Carle, ValérieDe La Varga, HerminiaPierzchalska, MałgorzataDe Lago, EvaPierzchalski, PiotrDe Lange, PieterPignatti, PatriziaDe Maagd, RuudPignochino, YmeraDe Martino, AndreaPiiper, AlbrechtDe Masi, LuigiPikuła, MichałDe Matteis, ValeriaPilarsky, ChristianDe Michelis, Maria IdaPillai, ShivDe Moerloose, PhilippePillaiyar, ThanigaimalaiDe Morais, Rui Manuel Santos CostaPilon-Smits, Elizabeth A. H.de Paiva, CintiaPilu, RobertoDe Pinho, Maria NorbertaPincus, SethDe Re, ValliPingault, LiseDe Rienzo, AssuntaPinheiro, MarinaDe Rosa, Maria CristinaPinho, Brígida RibeiroDe Santi, MauroPinkl, StefanDe Santis, RobertoPinto, AntonioDe Smaele, EnricoPinto, MafaldaDe Totero, DanielaPinto, MilenaDe Vecchis, RenatoPinzani, PamelaDe Verdal, HuguesPiplani, HonitDe Visser, SamuelPiqué, NúriaDe Vita, AlessandroPirici, DanielDe Vita, ValerioPirillo, AngelaDe Vries, CarliePiróg, Katarzyna A.De, KuntalPirola, LucianoDe, PradipPirtoli, LuigiDeaglio, SilviaPisarcik, MartinDean, AfshanPislariu, CatalinaDearnaley, John D. W.Pissarek, MargitDebeljak, NatašaPita, SebastianDeCoursey, ThomasPitt, SamanthaDeevska, GerganaPittalà, ValeriaDeFea, KathrynPittler, SteveDegiacomi, Matteo T.Pitto, LetiziaDegirmenci, VolkanPitucha, MonikaDegola, FrancescaPiva, RobertoDeissler, HeidrunPiva, TerrenceDel Gaudio, CostantinoPiya, SarbottamDel Genio, CharoPizzio, GastonDel Giudice, RitaPizzolanti, GiuseppeDel Mistro, AnnarosaPlacido, JerssonDel Re, BrunellaPlaczek, William J.Del Río Celestino, MercedesPla̧der, WojciechDel Rio, DanielePlanchais, SéverineDela Pena, IkePlano, DanielDelaloy, CelinePlante, IsabelleDelaney, ColmPlantier, LaurentDelannoy, PhilippePlastina, PierluigiDeleuze, HervePlatania, ChiaraDelhanty, PatricPlate, ManuelaDelihas, NicholasPlatt, Jeffrey L.Delisle, Jean-SébastienPlaza-Zabala, AinhoaDellavia, ClaudiaPłażek, AgnieszkaDelle Monache, SimonaPleasants, John M.Dellis, OlivierPloner, ChristianDello Ioio, RaffaelePlonka, PrzemyslawDelogu, GiovannaPłotka-Wasylka, JustynaDelogu, LuciaPlotnikov, Egor Y.Delorme, VincentPluquet, OlivierDemarquoy, JeanPluta, RyszardDemir, Ihsan EkinPniewski, TomaszDemirhan, ErhanPocheć, EwaDemyanets, SvitlanaPoggi, AlessandroDeng, Win-PingPoggio, PaoloDenis, SerenoPognonec, PhilippeDenisov, IliaPogoda, Hans-MartinDenko, NicholasPohlmann, StefanDeokar, HemantkumarPoku, Rosemary A.Deplazes, EvelynePolakowski, Nicholas J.Der Schaff, Arjen VanPolanska, KingaDeriu, Marco AgostinoPoławska, EwaDeRosa, MariaPoli, AlessandroD'errico, StefanoPołom, KarolDeryusheva, SvetlanaPommer, BernhardDesai, JigarPonnambalam, SreenivasanDesai, Varsha G.Ponomarev, EugeneDesando, GiovannaPons, AntoniDesantis, VanessaPons, Daniel GabrielDeshmukh, RupeshPons, MiquelDeshmukh, Umesh S.Pons, SebastianDeshpande, ShayuPonz-Sarvise, MarianoDesmet, TomPoon, IvanDesprés, CharlesPoór, MiklósDessain, ScottPop, OanaDesvergne, BéatricePopa-Wagner, AurelDettweiler, UlrichPope, ChadDeuss, PeterPoplawski, TomaszDevarajan, PriyadharshiniPopoff, Steven N.Devasundaram, SanthiPorollo, AlekseyDevesa, JesúsPorporato, Paolo E.Devine, Patrick J.Porras, AlmudenaDevireddy, AmithPortillo, Maria Del CarmenDevy, JérômePoŝa, MihaljDey, Bijan KPosadas, PilarDhakal, DipeshPoschenrieder, CharlotteDhamija, SonamPossidente, BernardDhanasekaran, Danny N.Possik, EliteDhariwal, RamanPosta, KatalinDharker, NachiketPotaczek, Daniel P.Dharmawardhane, SuranganiePoterszman, ArnaudDhawan, DeepikaPottosin, IgorDi Bartolomeo, AntonioPoudel, BarunDi Donato, MarziaPourcelly, GeraldDi Fazio, PietroPourquier, PhilippeDi Franco, ManuelaPowell, JoannDi Giulio, MassimoPowell, Jonathan J.Di Liegro, ItaliaPowrózek, TomaszDi Marcotullio, LuciaPozo Devoto, VictorioDi Napoli, AriannaPozo, Óscar J.Di Palma, MichaelPozzobon, MichelaDi Pierro, ProsperoPrabhu, K. SandeepDi Pietro, CinziaPrakasam, SivaramanDi Primo, CarmeloPrakash, AishwaryaDi Renzo, LiviaPratelli, AnnamariaDi Sansebastiano, Gian PietroPrats, Anne-CatherineDi Zazzo, ErikaPratsinis, HarrisDias, George J.Praud, ChristopheDíaz Rodríguez, IsabelPrearo, MarinoDiaz, FranciscaPrehn, JochenDiaz-Rodriguez, PatriciaPreissner, Klaus T.Dibb, NicholasPrentice, HowardDidiášová, MiroslavaPret, Anne MarieDidonna, AlessandroPreto, AnaDie, JosePrice, Jeffrey L.Dieckmann, Klaus-PeterPrice, PatriciaDiederich, MarcPrice, Ramona SalcedoDiekman, AlanPrickett, TimDiermeier, SarahPrieto, IsabelDijkhuizen, LubbertPrieto, ManuelDijkstra, Bauke W.Prieto-Lloret, JesusDillman, Adler R.Primig, MichaelDilokpimol, AdipholPrimikyri, AlexandraDiMattia, GabrielPrimus, Carolyn M.Dimitroff, Charles J.Prinsi, BhaktiDimmock, Jonathan R.Proestos, CharalamposDimopoulos, KonstantinosProfumo, ElisabettaDing, BaoqingProietti, PrimoDing, CherlynProkai, LaszloDing, JianxunProkesch, AndreasDing, LinProost, PaulDing, TianbingProvazník, IvoDing, ZhaoyangPrudent, MarionDings, Ruud P. M.Pruess, Birgit M.Dinh, Dzung H.Przybylska-Gornowicz, BarbaraDinischiotu, AncaPrzybyt, MałgorzataDinulescu, Daniela M.Psarra, Anna-Maria G.Dinwiddie, Darrell L.Psomas, GeorgeDiomede, FrancescaPsurski, MateuszDionisio, GiuseppePu, JingDioum, El Hadji M.Puca, AnnibaleDioum, Elhadji M.Pucciarelli, SandraDiPaolo, Richard J.Puchałka, RadosławDisdier, ClemencePucher, JohannesDistel, MartinPuglia, DeboraDiTacchio, LucianoPuglia, G.Dittmer, Neal T.Pugliese, GiuseppeDivi, Rao L.Puigdomènech, PereDixon, Dan A.Puiggalí, J.Dixon, David A.Puiggalí, JordiDixon, Ian M.C.Pulignani, SilviaDizdaroglu, MiralPunganuru, Surendra ReddyDkhar, Hedwin KitdorlangPunyadeera, ChamindieDluzen, DouglasPuri, AkshitDo, Duy NgocPurohit, Prashant K.Do, Sun HeePüschel, Gerhard P.Do, Yi-YinPutnam, William C. (Trey)Dobrikova, Anelia G.Pyka-Pająk, AlinaDobson, Renwick C. J.Pytka, KarolinaDocimo, TeresaQi, Robert ZhongDockrell, HazelQiang, ZheDoddapaneni, RaviQin, JuanDoddapattar, PrakashQing, LimingDodds, W. JeanQiu, HuanDoekes, GertQuarantelli, MarioDoi, KazuyaQuarck, RozennDolga, AmaliaQuartu, MarinaDoliana, RobertoQuattrocelli, MattiaDolzhenko, AntonQuesada Pérez, Víctor ManuelDombrovsky, AvivQuintana, JoséDomenicotti, CinziaRaake, Philip W. J.Domigan, Laura JoyRabanal Anglada, FrancescDomingo-Calap, PilarRabbani, NailaDomínguez Rebolledo, Álvaro EfrénRaber, JacobDomínguez-Álvarez, EnriqueRachek, LyudmilaDomozych, David S.Raczkowska, JoannaDonadel, GiuliaRadchenko, Eugene V.Donadelli, MassimoRadic, TanjaDonaire, LiviaRadoslaw Kajetan, KowalskiDong, LanfengRadu, Beatrice MihaelaDong, ShihuiRae, ColinDong, XiaoRafiei, HosseinDong, YiboRaggi, LorenzoDongiovanni, PaolaRagusa, AndreaDonkersley, PhilipRagusa, Maria AntoniettaDonnelly, DeirdreRahat, Michal A.Donners, MarjoRahib, LolaDonninger, HowardRahimi, FaridDonnini, SandraRahman Rashid, Rizwan AbdulDonno, DarioRahman, Md ToufiqurD'Onofrio, NunziaRahman, Md. MahbuburDonzelli, ElisabettaRahman, SafikurD'Orazio, JohnRahmanpour, RahmanDorey, C. KathleenRai, KrishanDormond, OlivierRai, PrakashD'Orsi, BeatriceRai, VikrantDos Santos, José C. S.Raimondi, LaviniaDos Santos, Reinaldo SousaRaimondo, StefaniaDosoky, NouraRaina, MedhaDosoky, Noura S.Raina, SatishDostmann, WolfgangRais, RanaDoudin, KhalidRaj, NitinDoulgeraki, AgapiRajakumar, GovindasamyDouros, Jonathan D.Rajala, PauliinaDovat, SinisaRajarathnam, KrishnaDovinova, ImaRaje, MithunDovizio, MelaniaRajendran, VazhaikkurichiDownie, J. AllanRajesh Lenin, RajiDowns, Diana M.Rajić, ZrinkaDoyle, HesterRajnarayanan, RajendramDoyle, SiamsaRajpurohit, SurendraDoyle, ToddRajurkar, MihirDr. Grant, WilliamRakitin, AlekseiDrago, FrancescoRam Kumar, Ram MohanDragomir, MihneaRamachandran, AnupDrancourt, MichelRamachandran, VinoyDrawert, BrianRamadori, GiulianoDrenjančević, InesRamalingam, NagendranDresch, JacquelineRamalingam, NaveenDrevet, RichardRaman, HarshDreyer, IngoRamanavicius, ArunasDreyfus, CherylRamani, VijayDriscoll, James J.Ramaraj, PanduranganDriver, JohnRamasamy, MohankandhasamyDrougard, AnneRamchandani, DivyaDrummond, RebeccaRamegowda, VenkategowdaDrysdale, ThomasRamich (Pivovarova), OlgaDu, CaiganRamil, CarloDu, DeguoRamos, CatarinaDu, WaRamsay, Michael A.Du, Xiao-junRana, DipakDu, XinRandall, Stephen K.Du, XingrongRangachari, ManuDuan, BinRangan, GopiDuarte, AndreiaRangel-Barajas, ClaudiaDuarte, José M.Rangnekar, AbhijitDuarte, Maria CristinaRanieri, GirolamoDubey, Mukesh K.Raniszewski, GrzegorzDubielecka, Patrycja M.Ranjan, AlokDubois, AnnickRanjkesh, AmidDubrez, LaurenceRanzato, EliaDuclot, FlorianRao, C. V.Duda, MałgorzataRao, VidhyaDudas, JozsefRapacz, MarcinDudnikov, AlexanderRapak, AndrzejDudok, JeroenRapisarda, VenerandoDuffy, Aaron M.Raposio, EdoardoDugé De Bernonville, ThomasRäsänen, KatjaDuh, Pin-DerRasche, LeoDuina, Andrea A.Raschi, EmanuelDumitru, Andra-CristinaRaspanti, MarioDun, Matthew D.Rastija, VesnaDungan, CoryRatajczak, EwelinaDunner, SusanaRatajewski, MarcinDunphy, Michael J.Ratan, AakroshDuong, HaiminhRatet, PascalDuranton, ChristopheRathinasabapathy, AnandharajanDurazzo, AlessandraRathinavelu, AppuDurek, ThomasRatkowsky, David A.Duryee, Michael J.Rato, Luís Pedro FerreiraDusi, VeronicaRatovitski, EdwardDutta, ArijitRattan, RamandeepDutta, ArnobRatz-Łyko, AnnaDutta, PalashRauch, BernhardDuwaerts, CarolineRauch, JensDvorakova-Hortova, KaterinaRautio, JarkkoDwinell, Michael B.Ravaioli, StefanoDwivedi, ChandraharRavegnini, GloriaDyakin, Victor V.Ravelonandro, MichelDyall, JulieRavichandran, AshwinDyr, JanRavuri, SudheerDzemidchyk, VadzimRawla, PrashanthDziedzic, ArkadiuszRawling, TristanDziegiel, PiotrRay, William C.Dziegielewska, BarbaraRayalam, SrujanaDziewit, LukaszRazaviyan, JavadDzimitrowicz, AnnaReader, Jocelyn C.Dziubla, ThomasReading, Benjamin J.Dzobo, KevinRedan, BenjaminEaton, DouglasReddy, SakamuriEbara, MitsuhiroRedell, MicheleEberini, IvanoRedmer, TorbenEbert, ThomasRedondo-Muñoz, JavierEbihara, LisaReed, Kevin F. M.Eble, JohannesReese, Benjamin E.Ecke, ThorstenReeve, VivienneEdamakanti, ChandrakanthRegal, JeanEdlich, FrankRege, ShraddhaEdwards, MartinRehfeld, JensEfferth, ThomasRehman, Junaid U.Efird, JimmyRehrig, Erin M.Efremenko, ElenaReinders, JoergEgawa, GyoheiReinhold, DirkEgger, BorisReis, HenningEhlen, J. ChristopherReiser, Peter J.Ehmer, UrsulaReisman, Scott A.Eichstaedt, ChristinaRemesh, SoumyaEid, NabilRen, DongmeiEiler, Daniel R.Ren, HongEinkauf, Jeffrey D.Ren, KaixuanEinspanier, RalfRen, ShuxinEisel, UlrichRena, GrahamEkberg, JennyRenaudineau, YvesEkielski, AdamRengaraj, DeivendranEkiert, HalinaRenshaw, Stephen A.Eklund, Erik A.Requicha, JoãoEl Aidy, SaharResch, UlrikeEl Bairi, KhalidRescifina, AntonioEl Kasmi, FaridReshkin, Stephan J.El Muayed, MalekRestivo, Francesco MariaEl-Bacha, TatianaReszka, EdytaElbasyoni, IbrahimReukov, VladimirEldeeb, MohamedReuter, KlausEleftheriadis, TheodorosRevelli, LucaEleftheriou, Eleftherios P.Reverberi, MassimoEleni, AnastasiadouRewatee, GokhaleEleonora, LeucciRey, LuisEl-Esawi, MohamedReyes, FernandoElferink, CornelisReynoird, NicolasEl-Gazzar, Mohamed M.Rezaei-Ghaleh, NasrollahElghobashi-Meinhardt, NadiaReznik, Jacqueline E.Elia, IlariaReznik, SandraEliopoulos, Elias E.Rezvani, KhosrowEl-Kadi, AymanRezzani, RitaElkon, RanRhee, InmooElleuche, SkanderRhee, Jin-KyuElli, StefanoRiar, Amanjot KaurEllsworth, BuffyRibas, GloriaEl-Naggar, AdelRibchester, RichardEl-Seedi, Hesham R.Ribeiro, ClarisseElshimali, Yahya I.Ribeiro, JoãoElson, AriRibeiro, LucasEmpl, MichaelRibeiro-Barros, Ana I.Emri, EszterRicardo, SaraEndo, MakotoRiccardi, CarloEnemark, EricRiccardo, Zenezini ChiozziEngel, AndrewRiccardo, ZoiaEngelberg, DavidRiccetti, LauraEngelberth, JurgenRicci, GiuliaEngelsdorf, TimoRicciardelli, CarmelaEnglezos, VasileiosRicha, TambiEnguita, FranciscoRichards, Lora A.Enguita, Francisco J.Richardson, JonathanEnos, Reilly T.Richly, HolgerEnrich, CarlosRichter, KatrinEnugutti, BalajiRichter, PeterEom, Gwang HyeonxRiemann, MichaelEppler, ElisabethRienzo, MonicaEpstein, Alan L.Riera, MarinaErb, RandallRiethdorf, SabineErceg, SlavenRiga, MariaErdmann, KatiRigano, Maria ManuelaErickson, MichelleRigas, StamatisErlandsson, Malin C.Rigó, GáborErnst, WolfgangRigoni, MichelaErny, DanielRikkerink, ErikErrington, Timothy M.Riley, Kathryn R.Ertaylan, GokhanRimbara, EmikoEscobar, CarolinaRimer, MendellEscobar, Matthew A.Rimondini, LiaEscobedo-Lucea, CarmenRinnerthaler, MarkEscola-Gil, Joan CarlesRiond, JoëlleEscudero, NuriaRiool, MartijnEskina, Erika NRiquelme, ErickEspada, JesúsRishi, ArunEspadas Villanueva, IsabelRissiek, BjoernEspel, EnricRissiek, BjörnEspen, LucaRiteau, BeatriceEsposito, CiroRitter, Christoph A.Esposito, Emilio XavierRitz, UlrikeEsposito, FrancescoRivas, CarmenEspuny, Antoni CaminsRivas, FatimaEsque, JérémyRivera, VictorEstavillo, GonzaloRivera-Vega, Loren J.Esteban, SusanaRivero, FranciscoEstelrich, JoanRivero, SoniaEstensoro, ItziarRivero-Pérez, M. DoloresEsteve-Romero, JosepRivilla, RafaelEsteves, IvaniaRizos, HelenEstevez, Jorge D.Rizvi, TahirEstévez, RaúlRizzarelli, EnricoEstour, FrançoisRizzo, Angela MariaEtalo, DesalegnRizzo, MilenaEubank, TimothyRizzolio, FlavioEufemi, MargheritaRizzolo, Lawrence J.Éva, CsabaRizzuti, BrunoEvans, GethinRo, HyunjuEves-van Den Akker, SebastianRobert Boisivon, HeleneEvrard, Solène M.Robert, ChristelleEzhov, Marat V.Roberts, SallyEzura, YoichiRoberts, SigridFabbretti, AttilioRoberts, WayneFabian, CarolRobertson, AvrilFabiani, RobertoRobertson, JeremyFabio Zuluaga, HectorRobin, François B.Fabris, LindaRobin, MacDiarmidFabris, LucaRobinson, CliveFabrizius, AndrejRobinson, DavidFacchiano, AngeloRobinson, KarenFagerstedt, KurtRobinsons, JameFaggio, CaterinaRoca, JoaquimFahraeus, RobinRocca, StefanoFailla, Cristina M.Rocha, SoniaFairweather, StephenRocha-Ferreira, EridanFaísca, PatríciaRochaix, Jean-davidFaiyue, BualuangRocha-Martin, JavierFakhoury, AhmadRochani, AnkitFalahati Anbaran, MohsenRocha-Rodrigues, SílviaFalcieri, ElisabettaRockel, Jason S.Falcone, GermanaRödel, FranzFalconer, RobertRodenbeck, Stacey DineenFales, AndrewRodolfo, CarloFalkinham, JosephRodrigo, IsmaelFan, ChenguangRodrigues, Celia F.Fang, JunnanRodrigues, JoaoFang, MingxiRodrigues, Márcia T.Fang, XiaolanRodrigues, MelanieFang, YiRodrigues, NunoFanizzi, Francesco PaoloRodrigues, OlivierFantini, JacquesRodriguez Lopez, Carlos M.Fantini, SebastianRodríguez, Alexander J.Fantuzzi, LauraRodríguez, AmaiaFarah, shadyRodríguez-Alvarez, JoséFare, SilviaRodriguez-Barbosa, José I.Fareleira, PaulaRodriguez-Cueto, CarmenFaria Albanese, Jimmy AlexanderRodriguez-Menocal, LuisFarnoud, AmirRodriguez-Saona, CesarFarolfi, AlbertoRoe, Daniel R.Farr, Christine J.Roelen, BernardFarrar, KerrieRoffi, AliceFarrar, MarkRogalska, AnetaFarrugia, BrookeRogers, Melissa B.Fasinu, Pius S.Rogozhin, EugeneFattuoni, ClaudiaRoh, SanghoFauque, PatriciaRohacs, TiborFauré, JulienRohila, JaiFazakerley, DanielRohira, AartiFebbraio, FerdinandoRojo, EnriqueFedele, FrancescoRojo, Maria AngelesFederico, AntonioRokavec, MatjazFedorova, Olga V.Rokosz, KrzysztofFehrenbacher, Jill C.Roldão, AntónioFeichtinger, GeorgRolfo, AlessandroFelgueiras, HelenaRom, SlavaFelix, Klaus M.Romanazzi, GianfrancoFelley-Bosco, EmanuelaRomani, AndreaFend, FalkoRomani, MassimoFeng, ChuangRomano, NiclaFeng, JingRomano-Chernac, Fabian B.Feng, Zhi ChaoRomei, CristinaFeo, FrancescoRomeo, GiovannaFeraco, AlessandraRomeo, RobertoFerchaud, Anne-LaureRomero, AlejandroFerens-Sieczkowska, MirosławaRomero, Fernando M.Feresin, Rafaela G.Romero, Marta R.Ferguson, BradleyRomero-Canelón, IsoldaFerguson-Miller, ShelaghRomero-García, Juan M.Fernandes, Maria HelenaRom-Jurek, Eva-MariaFernandez, Agustin F.Rommer, PaulusFernández, IgnacioRomo, Maria Del Mar Ortega-villaizanFernández, Irene GarcíaRon, ElioraFernández, José A.Ronan, KapetanovicFernandez-Alfonso, MariaRonca, AlfredoFernández-Ballester, GregorioRondanino, ChristineFernandez-Botran, RafaelRonellenfitsch, Michael W.Fernandez-Jimenez, NoraRonkainen, JustiinaFernández-Lafuente, RobertoRönö, KristiinaFernández-López, ManuelRooj, ArunFernandez-Martinez, LorenaRoot-Bernstein, RobertFernández-Mateos, PilarRopka-Molik, KatarzynaFernández-Ponce, M. TeresaRoques, Christian-FrançoisFernández-Ruiz, JavierRosa, ViniciusFernandez-Valle, CristinaRosado, Juan A.Fernando, DaniloRosato, AntonioFerrage, FabienRosellini, DanieleFerrante, AntonioRosenbluth, Jennifer M.Ferrante, ClaudioRosencrantz, Ruben R.Ferrante, PatriziaRosenthal, KenFerrari, PaolaRoseti, LiviaFerrario, ValerioRosicka-Kaczmarek, JustynaFerraz, RicardoRosołowska-Huszcz, DanutaFerreira, Paula M.Ross, IanFerreon, Allan Chris M.Ross, Ian L.Ferreri, CarlaRossa Jr, CarlosFerretti, ElisabettaRos-Santaella, José LuisFerri, Giovanni MariaRossignol, JulienFerrigno, AndreaRostaing, LionelFerris, WilliamRosti, VittorioFerrus, AlbertoRoterman, IrenaFestuccia, NicolaRoth, WilliamFigee, MartijnRothman, Alexander M.K.Figueiredo, AndreiaRotili, DanteFigueiredo, Marxa L.Rotllant, JosepFigueroa-Romero, ClaudiaRouached, HatemFilaire, EdithRoubelakis-Angelakis, KalliopiFilannino, PasqualeRouch, AlFilek, MariaRouhiainen, AriFilella, XavierRounbehler, Robert J.Filgueira, LuisRouse, Matthew N.Filhol, OdileRousseau, Anne SophieFiligheddu, NicolettaRousset, RaphaëlFilosto, MassimilianoRoussis, VassiliosFine, James BurkeRout, BhimsenFinelli, CarmineRoutledge, MichaelFinkemeier, IrisRovero, PaoloFinšgar, MatjažRoy Choudhury, GouravFinsterer, JosefRoy Choudhury, SwarupFiocchetti, MarcoRoy, AnuradhaFioravanti, AntonellaRoy, DipankarFiori Gradia, DanielaRoy, KasturiFiorito, SilvanaRoy, SonaliFiorotto, RominaRoy, SouravFirestein, BonnieRoychoudhury, AryadeepFischer, AndréRoyo, FelixFischer, NicoleRozhon, WilfriedFischer-Fodor, EvaRóżycki, BartoszFisher, ScottRua, DiegoFisslthaler, BeateRübe, ClaudiaFiszer, AgnieszkaRubio Valverde, LourdesFiume, GiuseppeRubio, VicenteFixen, KathrynRubis, BlazejFlack, JohnRucker, Robert B.Flavell, David J.Ruckert, FelixFletcher, Nicola F.Ruddraraju, Kasi ViswanatharajuFlorczyk, Stephen J.Ruden, DouglasFlores, Francisco B.Rudolph, BryanFloresta, GiuseppeRudrabhatla, SairamFlórez, Laura V.Rudykh, StephanFlorin, LuiseRudzitis-Auth, JeannetteFlorio, TullioRuelland, EricFlournoy, NikaelaRuggeri, FrancescoFogarty, GeraldRuggeri, PaoloFojt, LukasRuggerone, PaoloFojtová, MiloslavaRughoobur, GirishFolco, Hernan DiegoRuiu, LucaFolini, MarcoRuiz Lozano, JuanFölster-Holst, ReginaRuiz, MarioFonseca, AnaRuiz-Echevarria, Maria J.Fonseca, BrunoRuiz-Ederra, JavierFonseca, CarlaRuiz-Pérez, María VictoriaFonseca, FilomenaRund, DeborahFontana, LucaRuoppolo, MargheritaFontana, Robert J.Rupasinghe, ThusithaFont-Burgada, JoanRupenthal, IlvaFontes, PauloRupérez, Azahara IrisFoo, EloiseRupprecht, RainerForcales, Sonia-V.Rurek, MichalForget, AurelienRusciano, DarioForghieri, FabioRuscica, MassimilianoFormanek, PavelRusmini, PaolaFormisano, LuigiRuss, BrendanFornara, FabioRusso, AnnapinaForn-Cuni, GabrielRusso, AntonellaForneris, FedericoRusso, AntonioForrest, MarcRusso, GiuliaForster, BrittaRusso, MariaForsyth, Christopher B.Russo, NinoFort, Patrice E.Ruthstein, SharonForte, EleonoraRuzicka, JiriForte, ElviraRuzicka, KamilFortea-Verdejo, MartaRyabchikova, Elena I.Foster, TimRyan, Michael P.Foster, Warren G.Ryan, RobertFosu-Nyarko, JohnRybak, LeonardFouassier, LauraRytel, LilianaFountain, Samuel J.Ryu, Ji-KanFournier, NeilRyu, JongFourtounas, CostasRzepecki, RyszardFousteris, ManolisRzymowska, JolantaFox, ElizabethS. Douches, DavidFox, GlenSaad, HosamFrago, EnricSabater, BartolomeFrame, Leigh A.Sabirov, Ravshan Z.Francés-Soriano, LauraSaccà, BarbaraFrancisco, Javier López-BaenaSacchi, Gian AttilioFranco, Aime T.Saccone, ValentinaFranco, Albina Cristina RibeiroSachkova, MariaFranco, DiegoSacitharan, Pradeep KumarFranco, LuisSadeghi, SheilaFranco, OmarSadler, AnthonyFranco, RodrigoSaez, CarmenFrancolini, MauraSafavi-Hemami, HelenaFrancuz, TomaszSaferding, VictoriaFrank, SašaSagnou, MarinaFranken, Nicolaas A. P.Sagui, CelesteFrankenberg, StephenSaha, AchintoFranko, AndrasSaha, ShyamaliFraser, Christopher S.Saha, SuryaFrazzi, RaffaeleSahab, SareenaFrederix, P. W. J. M. (Pim)Sahara, NaruhikoFreedman, HollySahoo, PabitraFreeman, Linnea R.Sahoo, Pabitra KumarFreeze, Hudson H.Sahu, JagajjitFreire, JoaoSai, Kiran Kumar SolingapuramFrese, Steven A.Saielli, GiacomoFrieboes, HermannSaini, ShikhaFriml, JiriSainlos, MatthieuFrkanec, RužaSaisho, YoshifumiFroeyen, MatheusSaito, MarikoFroeyen, MathySaito, YoshiroFröhlich, EleonoreSaito, YukihiroFrolov, AndrejSaitoh, KenjiFromen, CathySaitoh, MasaoFrossard, Jean-PierreSaitoh, YasukazuFrugis, GiovannaSaiz-Fernández, IñigoFrungillo, LucasSakaguchi, MasakiyoFu, Shih-FengSakaguchi, YusukeFu, ShulanSakai, AtsushiFu, YangxinSakai, RyuichiFu, YuSakakibara, IoriFuchs, MarcSakamoto, KensakuFuehrmann, TobiasSakamuro, DaitokuFuentes-Mattei, EnriqueSaki, NajmaldinFujihara, HisakoSakkas, Lazaros I.Fujii, HiroakiSakr, SoulaimanFujii, TomomiSakuma, KunihiroFujii, YoshihiroSala, RobertoFujimori, AkiraSalaheen, SerajusFujimori, KoSalas, CarlosFujimoto, RyoSaleem, AyeshaFujita, MikakoSaleem, MuhammadFujita, MitsuguSalehin, MohammadFujita, SatoshiSaler, MarcoFujita, TetsujiSalgar, ShashikumarFujita, YuSalifoglou, AthanasiosFujita, YukiSalim, VonnyFujitani, YoshioSalminen, Juha-PekkaFujiwara, NaotoSalt, IanFujiyama, KazuhitoSalta, EvgeniaFukuchi, SatoshiSaluk-Bijak, JoannaFukuda, MachikoSalvarani, Nicolo'Fukui, HirokazuSalvarani, NicolòFukunaga, KenjiSalvatore, GiulianaFukunaga, TsukasaSalvatore, PepeFukushima, AtsushiSamaja, MicheleFukushima, TsuyoshiSambuy, YulaFukuta, ShiroŠamec, DunjaFuller, BryanSamhan-Arias, AlejandroFunaba, MasayukiSammi, ShreeshFunder, JohnSampathi, ShilpaFunel, NiccolaSamuels, IvyFunk, RichardSamuha, ShmuelFurler, Robert L.Samways, Damien S. K.Furukawa, TatsuhikoSan Fabian, EmilioFusaki, NoemiSan Jose, MichaelFutakuchi, MitsuruSanches Silva, AnaGaballa, SamehSánchez Pina, AmeliaGabelli, Sandra B.Sánchez-Fidalgo, SusanaGaber, TimoSanchez-Martinez, MelchorGaczynska, MariaSánchez-Purrà, MariaGaddam, Ravinder ReddySancho, AnaGafencu, AncaSanders, MatthewGagliano, TeresaSandow, Shaun L.Gagliardi, Paolo ArmandoSanejouand, Yves-HenriGahete, Manuel D.Sanghera, PaulGahlaut, VijaySangireddy, Sasikiran ReddyGaikwad, KishorSani, Marc-AntoineGaj, ThomasSanjust, EnricoGakiere, BertrandSankaran, Ganapathy SubramanianGalanis, AlexisSankaranarayanan, Nehru VijiGalateau Salle, FrançoiseSanmartín Grijalba, CarmenGalbiati, MassimoSanner, Michel F.Galderisi, UmbertoSano, NaotoGaletti, MariclaSansone, ClementinaGali, HimabinduSantacroce, LuigiGaligniana, MarioSantamaria, RitaGaligniana, Mario D.Santarelli, AndreaGáliková, MartinaSantarelli, LoryGalileo, Deni S.Santhakumar, AbishekGalindo, AntonioSantin, MatteoGalitzky, JeanSantini, EmanuelaGall Troselj, KoraljkaSantini, ValeriaGallant, NathanSantino, AngeloGallemí, MarçalSantofimia-Castaño, PatriciaGalleni, MorenoSantolla, Maria FrancescaGallo, CarmelaSantoni-Rugiu, EricGaluska, Sebastian P.Santos, Ana PaulaGalván, AuroraSantos, InêsGambino, GiorgioSantos-Gallego, CarlosGan, Ren-YouSantos-Ruiz, LeonorGanesan, LathaSantucci, AnnalisaGanetzky, Rebecca D.Santulli, GaetanoGangula, PanduSanz Garcia, AndresGanguly, AnutoshSanz-Clemente, AntonioGantenbein, BenjaminSanz-Herrera, Jose A.Gao, LongSaotome, MasaoGao, MinSaponaro, ChiaraGao, ShuaiSapudom, JiranuwatGao, TongchuanSaraiva, NunoGao, XiaolongSarapultsev, AlexeyGao, YangSarbini, ShahrulGao, YunxiangSaretzki, GabrieleGaponenko, VadimSargueil, BrunoGaraj, VladimírSarkar, ChandraniGaranto, AlejandroSarkar, DhrubaGarau, GianpieroSarkar, SibajiGarbayo, ElisaSarkes, Deborah A.García De Frutos, PabloSarnowski, TomaszGarcía Garrido, José ManuelSarti, Alba ClaraGarcia Vaquero, MarcoSasaki, HideoGarcia, IdoiaSasaki, Jun-IchiGarcía, NuriaSasaki, KazuhiroGarcía-Caballero, MelissaSasaki, MasanoriGarcia-Calderon, MargaritaSasaki, MotokoGarcía-Estañ, JoaquínSasaki, ReinaGarcia-Estañ, Luis PerezSase, AjinkyaGarcia-Gasca, Silvia AlejandraSassi, YassineGarcía-Hidalgo, JavierSatake, HironagaGarcía-López, Joan R.Satani, NikunjGarcía-Martínez, SantiagoSatbhai, SantoshGarcía-Martínez, TeresaSatbhai, Santosh BalasahebGarcia-Oliveira, Ana LuísaSathe, Gajanan JalindarnathGarcía-Ortega, LucíaSato, FuyukiGarcia-Pardo, JavierSato, HiromiGarcia-Redondo, Ana BelenSato, KenjiGarcia-Sosa, Alfonso T.Sato, KojiGarcia-Velasco, JuanSato, ShinGarcía-Villalón, Angel LuisSato, TakuichiGard, PaulSato-Bigbee, CarmenGardner, Paul D.Satoh, ShigeruGarg, Abhishek D.Satoh, TakayaGaribotto, ValentinaSatoh-Nagasawa, NamikoGarinis, GeorgeSatoshi, KameshimaGarner, Angelia D.Satou, RyousukeGarner, BiancaSattar, SampurnaGarofalo, MariangelaSau, SamareshGarrido-Cardenas, Jose AntonioSaunders, Nicholas A.Garrison, Jennifer L.Saurav, KumarGarrosa, ManuelSauter, DanielGascho, DominicSavaraj, NiramolGase, KlausSavary, StéphaneGaspar, DianaSavenkov, EugeneGasparri, FabioSavi, MoniaGasparrini, MassimilianoSavoie, Jean-MichelGasparyan, Armen YuriSavu, DianaGasse, CécileSaw, Constance L. L.Gaszner, BalázsSawada, HiroshiGatti, LauraSawada, ShojiroGaudet, D.Sawai, HirofumiGaudet, RachelleSawicka-Powierza, JolantaGauer, StefanSawicki, RafalGauld, James W.Scala, ValeriaGaumann, AndreasScalise, MariafrancescaGavaia, PauloScandurra, RobertoGavrilenko, TatjanaScarafoni, AlessioGavrilovic, JelenaScarel-Caminaga, R. M.Gawarecka, KatarzynaSchaap, FrankGdula-Argasinska, JoannaSchaberle, Till F.Ge, XiaodongSchäfer, WilhelmGeest, Bart DeSchaffer, Arthur A.Geffroy, ValerieSchäffner, AntonGeha, SamehSchaiquevich, PaulaGehlert, SebastianScheicher, Ralph H.Gehring, ChrisSchéle, ErikGehrmann, MathiasSchenk, MichaelGeibel, JohnSchepisi, GiuseppeGeiler-Samerotte, KerryScherer, GüntherGeng, FengSchiavone, MarcoGenova, TullioSchiffner, ReneGentile, FabrizioSchijns, Virgil E. J. C.Gentile, PiergiorgioSchild-Poulter, CarolineGentilini, DavideSchillaci, DomenicoGentilucci, LucaSchininà, Maria EugeniaGeorgakilas, AlexandrosSchinke, ThorstenGeorge Parsons, Kimberly SuzanneSchipper, Hyman M.Georgiades, SavvasSchirmer, BastianGeorgiev, VasilSchlachetzki, JohannesGeorgopoulos, NikSchlaepfer, Isabel R.Geraci, FabianaSchlessinger, AvnerGeraghty, PatrickSchlueter, Klaus-DieterGerbino, AndreaSchmidt, MarcGerdol, MarcoSchmidt, RebeccaGergs, UlrichSchmitt-Egenolf, MarcusGerhauser, IngoSchmitz, BorisGericke, MartinSchmitz, Hans-PeterGermain, PierreSchmoelzl, SabineGerman, DmitrySchmoll, DieterGerothanassis, Ioannis P.Schmutz, MarcGerratana, LorenzoSchneider, AnjaGerriets, ValerieSchneider, BarbaraGestal, Monica CartelleSchnichels, SvenGeszke-Moritz, MałgorzataSchoenhagen, PaulGetty, Kelly J. K.Schomacher, LarsGeurts, JeroenSchönberger, StefanGeurtsen, WernerSchönitzer, VeronikaGhaleh, BijanSchönthal, Axel H.Ghandhi, ShanazSchouten, AlexanderGhandhi, Shanaz AdiSchrock, MorganGhani Zadeh, HosseinSchroeder, ChristinaGhatak, ArindamSchroeder, HilkeGhislat, GhitaSchubert, MarioGhosh, ArnabSchuetz, AlexandraGhosh, SanchitaSchuhmann, Michael K.Ghosh, SantoshSchulte, CarstenGhoshal, KalpanaSchulz, FlorianGiachino, ClaudiaSchulz, MargotGiacomazza, D.Schulz, Wolfgang A.Giacomelli, ChiaraSchulze, GundulaGiacometti, JasminkaSchulze, JohannesGiakoustidis, Dimitris E.Schulz-Heddergott, RamonaGiampieri, FrancescaSchumacher, FabianGiangrande, AngelaSchutte, StaceyGianinetti, AlbertoSchwaminger, SebastianGiannakouros, ThomasSchwan, WilliamGiannelli, GianluigiSchwarz, Steven M.Gianni, PesSchweizer, MichaelGiannotti, Marina I.Schwelm, ArneGiannotti, Marina InésSchwen, DanielGiansanti, FrancescoSchwerdt, JulianGiaume, ChristianSchwertfeger, KayleeGibbs, Bernhard F.Schwiebs, AnjaGiesbertz, Pieter J.Sciacchitano, SalvatoreGil, KrzysztofScicchitano, PietroGilding, Edward K.Sciortino, Maria TeresaGiles, CoreyScorziello, AntonellaGiles, Wayne R.Scott, DanielGill, Martin R.Scotto D'Abusco, AnnaGilles, Maud-EmmanuelleScribante, AndreaGillet, ReynaldScrima, MarioGillevet, PatrickSebastian, AimyGilli, FrancescaSebastian, CarlosGimenez, EstelaSebastiana, MónicaGiménez, IgnacioSebastiani, FedericoGiordano, CarlaSebio, Ángel AbueloGiordano, NicolaSeca, Ana Maria Loureiro DaGiotta, LiviaSechi, GianPietroGiovannelli, PiaSeckinger, AnjaGiovannetti, ElisaSedic, MirelaGiovannini, AnnalisaSedio, Brian E.Giovannoni, RobertoSediva, AnnaGiraldo, PilarSedlačík, MichalGirao, HenriqueSeetharam, ArunGiraudo, EnricoSegura, AnaGiri, Sib SankarSegura, JoanGiron, Maria CeciliaSegura, JuanGiroud, MaudeSeibert, Franz JosefGiudice, JimenaSeidel, George E.Giuliano, MichelaSeifert, Georg J.Giuseppina, AndreottiSeiler, Magdalene J.Giusti, IlariaSeki, YoshinobuGizdavic-Nikolaidis, MarijaSękowski, SzymonGkolfakis, ParaskevasSelby, KatjaGłąbska, DominikaSeliger, CorinnaGlavas-obrovac, LjubicaSeligmann, HervèGloria, AntonioSelim, SamehGluba-Brzózka, AnnaSellayah, DyanGnocchi, DavideSellix, Michael T.Goberdhan, Deborah C. I.Semenyuk, PavelGoda, KeisukeSempere, Lorenzo F.Godos, JustynaSena, ArmandoGodzik, AdamSenatov, Fedor S.Goedegebuure, PeterSenbonmatsu, TakaakiGoedeke, LeighSendón, RaquelGoedert, JamesSengupta, ArjunGoel, PeeyushSengupta, DebrupGoffart, SteffiSenior, Alistair M.Gogal, Robert M.Seo, Hak SooGogulamudi, Venkateswara ReddySeoane-Collazo, PatriciaGoh, Boon ChongSepodes, B.Gohda, EiichiSerafini, GianlucaGöhre, VeraSerefko, AnnaGojo, SatoshiSergeant, KjellGok, OzgulSergi, ConsolatoGola, DamianSerio, GabriellaGolanov, Eugene V.Serralheiro, PedroGoldberg, Lynne J.Serrano, IreneGoldhawk, DonnaServis, MichaelGoldman, JeremySesaki, HiromiGoldman, Maria HelenaSessoms, Florence J.Goldring, MarySesti, FedericoGoldsmith, Jason R.Sestili, FrancescoGoldstein, Peter A.Seth, DevanshiGolenhofen, NikolaSeth, RataneshGollahon, LaurenSethi, GautamGoltsov, AlexeySethmann, IngoGolubkina, Nadezda AleksandrovnaSetzer, William N.Golubovskaya, VitaSeverson, Aaron F.Golz, John F.Seystahl, KatharinaGomaraschi, MonicaSgarzi, MassimoGombert, AlexanderSgrignani, JacopoGomes, SóniaShah, AjitGómez De Cedrón, MartaShah, DilipGomez Roldan, Maria VictoriaShah, ManishGomez, NidiaShah, RuchiGómez, RodolfoShahid, Muhammud QasimGómez-Baena, GuadalupeShanjani, YaserGómez-Florit, ManuelShanmugam, AvinashGomez-Lopez, NardhyShapiro, Bruce A.Gómez-Muñoz, AntonioSharan, RitiGomez-Pinilla, Pedro J.Sharif, ArianeGómez-Puertas, PaulinoSharma, AmrishGómez-Ruiz, SantiagoSharma, ArishyaGómez-Zorita, SaioaSharma, ArunGonçalves, Lidia M. D.Sharma, AtulGondi, Christopher S.Sharma, BheshGonec, TomasSharma, DeepikaGongol, BrendanSharma, NeeruGongora Castillo, ElsaSharma, RohitGonkowski, SlawomirSharma, ShwetaGonzales, Cara B.Sharma, SudhaGonzalez Bosc, Laura V.Sharma, SunnyGonzález, Florenci V.Sharman, Edward H.Gonzalez, GabrielShavandi, AminGonzález, María EugeniaShaw, LisaGonzalez, Michael J.Shcherbik, Natalia V.Gonzàlez-Duarte, RoserShearrer, Grace E.Gonzalez-Escamilla, GabrielSheen, Shyn-ShinGonzález-García, IsmaelShehata, NaderGonzález-González, RogelioShehu, AmardaGonzález-Hernández, TomásSheldrake, HelenGonzález-Navarro, HerminiaShelke, GaneshGonzález-Vallinas, MargaritaShen, Jia ZackGonzalez-Vicente, AgustinShen, ShengrongGoodier, Martin R.Shen, WeiranGopalakrishnan, GopanShen, YiGöpel, SvenShen, Yi-PingGorantla, VijayShen, Yu-FangGordiichuk, PavloSheng, RuilongGordon, DonnaSherbenou, Daniel W.Goreham, ReneeSheri, MadhuGorgey, Ashraf S.Sherr, David H.Gori, TommasoSheth, SandeepGorovits, BorisSheu, Ming-jyhGorrell, RebeccaShi, HaifeiGórska, SabinaShi, HuaGorski, JeffreyShi, JianGosselet, FabienShi, JiaqiGoswami, NanduShi, ZhiGoto, KatsumasaShiao, Young-JiGötte, MartinShibuya, HiroshiGötz, WernerShichita, TakashiGoukassian, DavidShiga, TakashiGould, GwynShigeishi, HideoGouveia, SusanaShigeru, HishinumaGovaere, OlivierShigeru, YatohGow, Megan L.Shih, Yu-RuGoyal, RajniShiina, TakashiGrabacka, MajaShikama, YosukeGrabauskas, GintautasShim, HyunboGrabinska, KarionaShim, Joon W.Grabsztunowicz, MagdaShim, RosalynGracia-Sancho, JordiShim, Yhong-heeGradilone, Sergio A.Shimada, MasakoGradinaru, Andrei CristianShimada, TsutomuGrafi, GideonShimada, YasuhitoGraler, Markus H.Shimizu, TakahikoGramates, L. SianShimizu, TakatsuneGramazio, PietroShimo, TsuyoshiGramlich, MichaelShimodaira, ShigetakaGramss, GerhardShimura, HanakoGrande Tovar, Carlos DavidShinde, SuhasGrandemange, StéphanieShingu, TakashiGrant, MichaelShioda, NorifumiGrässel, SusanneShiomi, DaisukeGrasselli, ElenaShiozawa, YusukeGray, Gordon R.Shipley, PaulGray, StevenShirahata, TatsuyaGrazul-Bilska, AnnaShiraishi, TakehikoGreeley, Siri Atma W.Shirasuna, KoumeiGreen, MichaelShirato, HirokiGreenberg, Richard N.Shivanna, BinoyGreenberg, Robert M.Shojaei, ShahlaGreene, CatherineShortt, JakeGreene, Lesley H.Shosha, EsraaGreenhalgh, Andrew DavidShostak, AntonGreenhalgh, DavidShowalter, AllanGreenwood, Michael T.Shu, JiangGregg, RandalShu, LongfeiGregor, IngoShukla, HemGregory, StephenShults, Elvira E.Greither, ThomasShunmugam, ArunGremmels, HendrikSiatka, TomášGresnigt, Mark S.Sicinski, RafalGresshoff, Peter M.Siddappa, ByrareddyGrether-Beck, SusanneSidhu, RohiniGrewal, Raji PaulSidorenko, Viktoriya S.Grewal, ThomasSidransky, EllenGriesbeck, AxelSié, PierreGriffin, RobertSieber, FritzGrignani, GiovanniSiegel, Sue RutherfordGrijalvo, SantiagoSiemer, Ansgar B.Grimholt, UnniSiemianowski, OskarGrimm, Marcus O. W.Sier, Cornelis F.M.Grinberg, DanielSierecki, EmmaGrisoni, FrancescaSignore, GiovanniGrix, TobiasSigusch, Bernd W.Grizzi, FabioSikavitsas, VassiliosGroban, LeanneSil, PayelGrochans, ElzbietaŠiler, BranislavGrolli, StefanoSillence, DanGrones, PeterSilman, IsraelGrosell, Martin H.Silva, Adriana RibeiroGross, AaronSilva, Ana M.Großhans, JörgSilva, LuísGrossi, GiancarloSilva, Margarida F. B.Gruca, AleksandraSilva, Nuno A.Grudnik, PrzemyslawSilvagno, FrancescaGruengreiff, KurtSilvan, UnaiGrumet, RebeccaSilvente-Poirot, SandrineGrumezescu, Alexandru MihaiSilvestre, SamuelGrunig, GabrieleSilvestri, IdaGruntenko, NatalySim, Sheina BGrzebelus, DariuszSimakov, S. S.Grzmil, PawełSimard, AlainGrzybowska, EwaSimard, MarcGrzymajlo, KrzysztofSimecka, Jerry W.Gu, FengŠimić, GoranGu, YuchaoSimitzi, CharaGualtieri, PaoloSimitzis, PanayiotisGuan, YuanŠimko, FedorGuardado-Calvo, PabloSimm, StefanGuariglia, EmanuelSimmen, Rosalia C. M.Gubbels Bupp, MelanieSimo, LadislavGubin, DenisSimões, Marta FilipaGudey, Shyam KumarSimon, IstvanGudmundsdottir, Bjarnheidur K.Simone, Angela DeGuéguen, LaurentSimonescu, Claudia MariaGuelcher, Scott A.Simoni, JanGuerlesquin, FrancoiseSimón-Mateo, CarmenGuerra, CarmenSimonsen, Henrik ToftGuerra, IvánSimon-Yarza, TeresaGuerra-Rebollo, MartaSimopoulou, MaraGuerrera, M. C.Simpson, David A. C.Guerrieri, EmilioSimpson, Julie E.Guha, Prajna P.Sims-Robinson, CatrinaGuha, SoniaSimurda, TomasGuhathakurta, SubhrangshuSingh, AbhishekGuibert, ChristelleSingh, AmareshwarGuiliani, NicolasSingh, AnilGuillermet Guibert, JulieSingh, AnuragGuinane, CaitrionaSingh, ArunimaGuiot, CaterinaSingh, Ashok K.Guix, Ester BallanaSingh, Brijesh KumarGuizzetti, MarinaSingh, ChandraGullberg, DonaldSingh, ChanpreetGulumian, MarySingh, Harminder PalGundamaraju, RohitSingh, JugpreetGunderson, Mark P.Singh, KamalendraGundogdu, OzanSingh, KhushwantGünes, CagataySingh, Kumar SaurabhGunness, PurnimaSingh, NarinderGünther, AndreasSingh, Naveen KumarGuo, Chih-HungSingh, RakshaGuo, LongbiaoSingh, ShaktiGuo, QiuquanSingh, SonalGuo, ShengfengSingh, VishalGuo, WeiminSingh-Cundy, AnuGuo, YinSingla, BhupeshGupta, DorinSinha, AmitGupta, Sheeba VargheseSinha, KalyanGupta, ShuchiSiniscalco, DarioGupta, VijayalaxmiSinyukov, AlexanderGupta-Rossi, NeetuSionov, EdwardGurevich, Vsevolod V.Sips, PatrickGurruchaga, MarilóSireci, GuidoGururani, Mayank AnandSiristatidis, CharalamposGust, RonaldSirko, SwetlanaGutiérrez, Norma C.Sirotkin, Alexander V.Gutiérrez-Juárez, RogerSirtori, CesareGutleb, ArnoŞişecioǧlu, MeldaGutschner, TonySita, GiuliaGuy, Charles L.Sitarek, PrzemysławGuzik, UrszulaSitcheran, RaquelH Shirvani, ArashSitz, Rachael A.H. Bailey, WilliamSivakumar, SushamaHa, HyunjungSivaprakasam, SathishHa, JungminSivasubramaniyan, KavithaHa, PatrickSiwek, AgataHaarmann-Stemmann, ThomasSixou, SophieHaase, HajoSizochenko, NataliaHachiya, TsuyoshiSizun, ChristinaHack, EthanSjöberg, FolkeHada, MegumiSkaaby, TeaHadden, KyleSkalsky, RebeccaHadjiargyrou, MichaelSkendros, PanagiotisHadjikakou, SotirisSkiba, MohamedHadjipavlou-Litina, DimitraSkirtach, AndreHadler-Olsen, ElinSkoneczny, MarekHaendler, BernardSkopouli, Fotini NikolaosHaffner-Luntzer, MelanieSkorik, Yury A.Häfner, NormanSkórzyńska-Dziduszko, Katarzyna E.Hagland, Hanne R.Skowronska, AgnieszkaHagstrom, Stephanie A.Skriver, KarenHailfinger, StephanSkurla, CarolynHajjar, KatherineSlamova, KristynaHakkinen, LariSlater, ClarkeHalatsch, Marc-EricSlawson, ChadHaldar, SumantoŚliwka, JadwigaHales, Karen H.Sloan, Kenneth B.Halford, MichaelSlominski, AndrzejHallows, KennethSlootweg, Erik J.Ham, DanielSmagghe, GuyHamacher, JürgSmall, Leo J.Hamanishi, JunzoSmani, YounesHambley, TrevorSmertenko, AndreiHamblin, MichaelSmiesko, MartinHametner, BernhardŚmieszek, AgnieszkaHamilton, Duane H.Smit, LindaHamm-Alvarez, SarahSmith Callahan, Laura A.Hamm-Alvarez, Sarah F.Smith, Brittany L.Hamon, MorganSmith, Hugh A.Hamrick, Mark W.Smith, JessicaHan, Byung WooSmith, Jill P.Han, DongSmith, KimberlyHan, Dong-WookSmith, MatthewHan, Hong-YuSmith, Micholas DeanHan, JaehongSmith, Patrick J.Han, PingpingSmith, Stephanie J.Han, QifengSmolikova, GalinaHan, XiaoyuanSmyth, JamesHanai, Jun-ichiSmyth, RedmondHanaoka, MitsumasaSnegur, LubovHandzlik, JadwigaSnowden, TimothyHanif, MuhammadSoares, Claudio M.Hankinson, OliverSoares, PaulaHano, ChristopheSobeh, MansourHano, TakuzoSobotta, LukaszHanson, JohannesSoe, KentHanss, MichelSoenen, Stefaan J.Hantusch, BrigitteSofronov, GeorgyHao, JiaqingSohl, ChristalHaque, InamulSokolove, JeremyHardiman, GerardSolano, FranciscoHarding, KatharineSoler, Miguel ÁngelHardingham, Jennifer E.Solomon, MelaniHardwick, James P.Solovyev, AndreyHardy, JohnSommariva, MicheleHardy, John G.Sommerhalter, MonikaHardy, RowanSon, Deok-sooHargreaves, IainSon, HokyoungHargreaves, Iain P.Sonda, SabrinaHariharan, SeethalakshmiSong, ChunhuaHarijith, AnanthaSong, GuoqingHarmanci, Arif OzgunSong, JeongminHarn, Horng-JyhSong, Jia L.Haro, DiegoSong, JianchengHarp, TylerSong, Lu-JieHarper, AlanSong, ShijieHarrington, CharlesSong, SuHarris, Andrew R.Song, Sung HoHarris, Edward N.Soni, DheerajHarris, WilliamSonkoly, EniköHarrison, Jonathan S.Sonntag, KaiHartman, MariuszSoole, KathleenHartman, MatthewSoós, VilmosHarvey, AdamSophia, ChernikovaHarvey, AlexandraSorg, OlivierHarvey, KirstenSoriano, Jose MiguelHarvill, EricSorrell, J. MichaelHasanuzzaman, MirzaSorrenti, ValeriaHasegawa, MorifumiSorrentino, ElenaHashimoto, MakotoSosna, JustynaHashimoto, MasayukiSoteriou, GeorgiosHaspel, NuritSottnik, JosephHassan, MohamedSouček, P.Hassan, Sherif T. S.Soucy, ShannonHatami, AsaSoudeyns, HugoHatano, NoriyukiSoulika, AthenaHatano, TsutomuSounni, Nor EddineHatt, SéverinSousa, SandraHattori, ToshioSousounis, KonstantinosHatziantoniou, SophiaSouth, AndrewHaudecoeur, RomainSouza-Fonseca-Guimarães, FernandoHaumann, MichaelSoverini, SimonaHaurani, Mounir J.Sowa, IreneuszHauser, PeterSowndhararajan, KandhasamyHausman, Gary J.Spagnuolo, RoccoHausner, GeorgŠpanič, ValentinaHawinkels, LukasSpassov, SashkoHayakawa, TohruSpegazzini, NicolasHayakawa, YokuSpégel, PeterHayashi, YoheiSpencer, Charles T.Haynes, Nicole M.Spencer, ThomasHazak, OraSpengler, DietmarHazawa, MasaharuSperanza, GiovannaHe, AinaSpiekerkoetter, EddaHe, ChunboSpinazze, AndreaHe, FeiSpingler, BernhardHe, FengSpizzo, RiccardoHe, GuanglongSpring, KevinHe, HailunSpyrakis, FrancescaHe, JingSquire, JohnHe, XiaoboŠrámek, JanHeaphy, ChristopherSreedasyam, AvinashHeath, TimothySridhar, JayalakshmiHeberden, ChristineSripathy, SmithaHebert, TerrySriram, GopuHeckathorn, Scott A.Srivastava, AkhilHegele, Robert A.Srivastava, AtulHeggermont, WardSrivastava, SaurabhHeggeset, Tonje Marita BjerkanSrivatsan, MalathiHegyi, PéterSrivenugopal, KalkunteHehlgans, StephanieSroka, ZbigniewHehlmann, RüdigerSt John, James A.Heiker, John T.St. Hilaire, CynthiaHeinbockel, ThomasStacchiotti, AlessandraHejatko, JanStachowska, EwaHelaly, SoleimanStafford, JamesHelle, FrancoisStahl, FelixHeller, GerwinStahl, FrankHeller, JanoschStaines, KatherineHellmann, Jason L.Stakenborg, NathalieHellström, MatsStam, RemcoHellweg, ChristineStańczyk, Wlodzimierz A.Helm, RichardStanek, AgataHelmy, AdelStange, KatjaHenagan, Tara M.Stanley, Jone A.Hendriks, LizzaStarodubova, ElizavetaHenen, Morkos A.Starowicz, MałgorzataHengesbach, MartinStarr, AmandaHenkel, JaninStasiak, AndrzejHennequin, ClaireStasko, JanHenriksson, MarieStathopulos, PeterHenrique, RuiStaunstrup, Nicklas HeineHeo, Yong-SeokStecca, BarbaraHer, Lu-ShiunSteen, IdaHerfindal, LarsSteenbergen, RenskeHerington, Jennifer L.Stefani, AmbraHermey, GuidoStefani, LauraHernández Ledesma, BlancaStefanovic, BrankoHernández, M. LuisaSteffan, Joshua J.Hernandez, MariaSteger, GertrudHernández-Hernández, ÁngelSteinhoff, U.Herold, NikolasSteinle, JenaHerr, Deron RaymondSteinmeyer, JuergenHerránz, HéctorStenbeck, GudrunHerrero-Beaumont, GabrielStendahl, OlleHerrmann, HaraldStephan, HolgerHertegård, StellanStepien, EwaHerten, MonikaStępień, ŁukaszHess, AngelaStepnik, MaciejHetman, MichalStevens, Joanne M.Heumann, RolfStevenson, BradleyHeusinkveld, HarmStewart, BeverlyHevey, RachelStewart, DelishaHewett, SandraStewart, JasonHeymann, DominiqueStewart, Michael W.Higashida, HaruhiroStieber, JulianeHihara, YukakoStieger, BrunoHildenbrand, GeorgStilli, DonatellaHildreth, BlakeStine, Keith J.Hilker, MonikaStjohn, JustinHillman, A. RobertStobdan, TseringHills, RonaldStobiecka, MagdalenaHimeno, HyoutaStobiecki, MaciejHimoto, TakashiStocco, CarlosHinderlich, StephanStojko, JerzyHindra, HindraStoleru, ElenaHinds Jr., Terry D.Stolfi, CarmineHinojosa, SilviaStopeck, Alison T.Hiraga, ToruStopka, TomášHirakawa, HidehikoŠtorchová, HelenaHirano, KatsuyaStorkus, WalterHirano, ShigeruStorlazzi, AuroraHirasaka, KatsuyaStorti, PaolaHirasawa, NoriyasuStossi, FabioHirata, YokoStournaras, ChristosHirata-Tsuchiya, ShizuStout, David A.Hirayama, TakashiStout, MichaelHiroi, NoboruStout, RandyHirsch, Mark A.Stow, JennyHirschi, Kendal D.Stoychev, GeorgiHiruma, KeiŠtrac, Dubravka ŠvobHo, HonhingStrasser, RichardHo, MengfeiStratmann, BerndHo, RogerStratmann, JohannesHo, Roger Chun ManStraus, Suzana K.Ho, Shin-LonStrauss, LauraHo, VincentStrauss, OlafHo, Wen-FuStrauss, PhyllisHo, Wing ShingStrauss, SarahHo, Yi-ChengStrekalova, TatyanaHoang, Nam V.Streubel, JanaHoang-Vu, CuongStriker, RobHöckner, MartinaStroffekova, KatarinaHodge, AllisonStrosznajder, Joanna B.Hodges, LynetteStrube, JochenHodson, MartinStrudwick, XantheHoffman, Nolan J.Struga, MartaHoffmann, KlausStubbs, BriannaHofmann, JohannStudzińska-Sroka, ElżbietaHøivik, Erling AndreStudzinski, George P.Hojjat-Farsangi, MohammadStührwohldt, NilsHojo, HironoriStupka, NicoleHolalu, SrinidhiSturlese, MattiaHolan, VladimirStylianou, AndreasHolecek, MilanSu, MengHolefors, AnnaSubhi, YousifHollenbach, MarcusSubramani, MayavanHollomon, Mario G.Subramaniam, PrasadHolmes, Dawn E.Subramanian, VeedamaliHolmøy, TrygveSud’ina, Galina F.Holst, OttoSuda, KenichiHolt, Scott M.Sudheeran, Pradeep KumarHolvik, KristinSudhir, Putty ReddyHolzel, ChristinaSudhir, Putty-ReddyHolzmann, KlausSugase, KenjiHoma, JoannaSugawara, AkihiroHomma, HiroshiSugimoto, NaotoshiHomma, TetsuyaSugimura, HaruhikoHong, QiutingSugiura, DaisukeHong, SoyonSugiura, YukiHong, YonggeunSuh, Dae-yeonHooda, JagmohanSuh, Won HyukHooykaas, Paul J. J.Suhail, YasirHopkins, SamanthaSuidan, GeorgetteHorejs-Hoeck, JuttaSuk, Ki TaeHorhat, Florin GeorgeSukhov, VladimirHori, ToshiyukiSukocheva, OlgaHori, YuuichiSukumaran, SunithaHorino, YoshikazuSulatskaya, AnnaHorn, Patrick J.Sulek, KarolinaHornsveld, MartenSuliburska, JoannaHorowitz, MiaSullivan, ChrisHorstkorte, RüdigerSullivan, Katie E. O’Horvath, David P.Sullivan, LucasHorvath, DragosSultan, FarazHorvath, RobertSuman, ShubhankarHosek, PetrSun, ChangpoHosgood, SarahSun, ChengwenHoshi, AkihikoSun, ConroyHoshiba, TakashiSun, FengjieHoshino, MasaruSun, JieHoskins, ClareSun, JingjingHosohata, KeikoSun, JunHosokawa, YoshitakaSun, LuHosório, HugoSun, MingkuanHossain, MokarramSun, QianHošťálková, AnnaSun, QingHotta, Jun-ichiSun, WeiHotta, YoshihiroSun, WeijingHou, AnfuSun, WenyueHou, Chien-WeiSun, WujinHou, HarveySun, XiaomingHou, PanpanSun, XinghuiHouen, GunnarSun, YingjieHowell, ViiveSunami, YoshiakiHoy, AndrewSundar, ReshmaHoyos-Villegas, ValerioSundaram, UmaHozbor, DanielaSundaramoorthy, PasupathiHrabal, RichardSung, SibumHrelia, PatriziaSunna, AnwarHsia, Shih-MinSunohara, YukariHsiao, GeorgeSunseri, FrancescoHsieh, ChengyingSuntharalingam, KogularamananHsieh, Chia-ChienSupernat, AnnaHsieh, Hsi-LungSurapathrudu, KanakalaHsieh, Tusty-JuanSuravajhala, PrashanthHsu, Chung Y.Surdacki, AndrzejHsu, Fang RongSurguchov, AndreiHsu, Fei-TingSuryawanshi, VasantikaHsu, Kuo-ShengSusic, MichaelHsu, Ren-JunSutaria, DhruvitkumarHsu, Shang-Te DannySutherland, John B.Hsu, Sung-PoSuzuki, HideoHsu, ToddSuzuki, HiroetsuHsu, Tsung-ISuzuki, Hiroshi I.Hsu, Yi-ChiungSuzuki, KatsuhikoHsueh, Yi-HuangSuzuki, TakafumiHu, GuangSuzuki, YukiHu, GuangganSvedas, VytasHu, HelenSvob Strac, DubravkaHu, JianyingSwamy, GaneshHu, Jin-JiaSwanberg, MariaHu, Marian Y.Swarbreck, Stéphanie M.Hu, MinSwayne, Leigh-AnneHu, QiwenSwerdlow, Russell H.Hu, ZhenbinSwevers, LucHuang, ChunfaSwiatkowska-Warkocka, ZanetaHuang, Guan-JhongSwierczynski, JulianHuang, HaoSyed, ViqarHuang, Huey-ChunSykes, PamelaHuang, Jason C.Sykora, PeterHuang, JianSymkal, PetrHuang, LeiSymonova, RadkaHuang, Lei (Helen)Szabados, LászlóHuang, Nai-KueiSzablewski, LeszekHuang, RongfengSzafranski, KrzysztofHuang, Shu-HungSzalay, Aladar A.Huang, WeiSzaleniec, MaciejHuang, Wen-LiiSzatmári, TündeHuang, WenruiSzczepanski, CarolineHuang, XiaohuSzczolko, WojciechHuang, YadongSzebeni, Gábor J.Huang, Ying-HsienSzeiffova Bacova, BarbaraHuang, Yu-ChuenSzeliga, MonikaHuang, Yung-FenSzéll, MártaHuber, HeinrichSzewczuk, Myron R.Huber, Stephan M.Szewczyk, KatarzynaHuber, VeronicaSzewczyk, Nathaniel J.Hubková, BeátaSzklarz, GrazynaHübner, ChristianSzopa, AleksandraHuchzermeyer, BernhardSztanke, MałgorzataHudzieczek, VojtechSztuba-Solinska, JoannaHui, Kwai FungSzweda, PiotrHui-Yuen, Joyce S.Szymanowska, UrszulaHumbeck, KlausSzymańska, RenataHumphries, BrockTabata, YasuhikoHumphries, Jonathan D.Tabish, TanveerHung, Shih-YaTacconelli, StefaniaHunter, Richard G.Tadashi, WatabeHunter, Robert P.Taddei, Maria LetiziaHuppi, KonradTadepalli, SirimuvvaHur, YoonKangTadesse, WuletawHura, KatarzynaTadych, MariuszHura, TomaszTafuri, AgostinoHurley, MargaretTaghibiglou, ChangizHuss, FredrikTaglietti, Angelo MariaHussain, MulazimTaguchi, AyumuHussein, WaleedTaguchi, KumikoHutchinson, EdwardTaguchi, Y-h.Hutchinson, Edward C.Taguchi, YoichiroHutchison, Robert E.Tahimic, CandiceHutmacher, Dietmar W.Tai, TCHwa Soung, YoungTai, WanboHwang, In KooTaira, JunseiHwang, Pung PungTajima, TsuyoshiHwang, YongsungTajiri, NaokiHyndman, Kelly AnneTakabe, WakakoHyun, Kyung-YaeTakahara, TerunaoHyun, Sang-HwanTakahashi, AkihisaI Gossmann, ToniTakahashi, HirokiIaccino, EnricoTakahashi, KenIacobazzi, VitoTakahashi, NobuyukiIacoviello, MassimoTakahashi, OhgiIannuzzi, ClaraTakahashi, ShinyaIatsenko, IgorTakahashi, TakuIbarra Molero, BeatrizTakahashi, ToshioIbrahim, HussameldinTakaki, AkinobuIbrahim, Sulaiman SadiTakaki, ManabuIchihara, SahokoTakakura, NobuyukiIchihashi, MasamitsuTakamatsu, DaisukeIchitani, KatsuyukiTakamatsu, ShigeyukiIde, KazukiTakano, KatsuraIdo, YasuoTakasawa, ShinIentile, RiccardoTakaya, JunjiIeraci, AlessandroTakayama, KenichiIgaz, PeterTakayama, YoshiharuIglesias-Fernández, RaquelTakebayashi, Shin-ichiroIglesias-Linares, AlejandroTakegahara, NorikoIguchi, TaisenTakeishi, YasuchikaIida, HiroshiTakemori, HiroshiIijima, NoriakiTakemoto, HiroyasuIizuka, KatsumiTakeuchi, TakeshiIkarashi, NobutomoTakeuchi, TomoharuIkeda, MasahiroTakó, MiklósIkegaya, NaokiTakumi, ShigeoIkeguchi, MasamichiTaleb, NadineIkehara, KenjiTallerico, RossanaIkeshima-Kataoka, HirokoTalukder, PoulamiIkura, YoshihiroTamaki, TetsuroIkushiro, Shin-ichiTamama, KenichiIkuta, TetsuroTamamis, PhanouriosIlagan, Ma. Xenia G.Tamarat, RadiaIlardi, GennaroTamarit, JordiIlavenil, SoundharrajanTamma, GraziaIlies, Marc A.Tammimies, KristiinaIlieva, ZhenyaTamura, YukinoriIlmer, MatthiasTan, C. SabrinaIlyas, AzharTan, Dun-XianIm, Chang-NimTan, Ek HanImai, HirooTan, JoanneImai, ToshioTan, Shyh-HanImai, YoichiTanabe, KatsuyukiImamoto, NaokoTanabe, KenjiImamoto, YasushiTanaka, HiromitsuImbert, VéroniqueTanaka, MasatoImbrici, PaolaTanaka, NobuyukiImhof, PetraTanaka, ShigeoImig, JohnTanaka, ShinjiImokawa, GenjiTanaka, TakujiImpellizeri, JosephTanaka, ToshiakiImperlini, EstherTanaka, YasuoIna, KenjiTanaka, YoshiakiInaba, HiroshiTanaka, YoshioInada, NataliaTang, LipingInagaki, TakeshiTang, Ming-JerInamura, KentaroTang, RenjieIncitti, TaniaTang, YouhongIndiveri, CesareTang, YuanIngre, CarolineTani, AkioIniesta, RaquelTaniguchi, HiroakiInoue, AtsukoTaniguchi, KoheiInoue, KazushiTanimizu, NaokiInui, AkioTankiewicz-Kwedlo, AnnaInui, MasafumiTanner, EdenIohara, DaisukeTanner, NathanIommarini, LuisaTanoi, KeitaroIordanskii, AlexeyTanonaka, KouichiIorio, Ronald M.Taoka, YousukeIorizzo, MassimoTaoufik, EraIotti, MircoTappia, Paramjit S.Iqbal, KhalidTaran, ElenaIqbal, Tariq H.Tarantini, StefanoIraci, NunzioTarantino, GiovanniIrato, PaolaTaraschi, Susanna MariaIriepa Canalda, IsabelTarasova, Nadya I.Irimura, TatsuroTariq, MohammadIrvine, Scott A.Tarozzi, AndreaIrving, Thomas C.Tarro, GiulioIsaji, ShujiTashima, ToshihikoIsaza Martínez, José HipólitoTashiro, HirotakaIsemura, MamoruTata, Ada MariaIsgaard, JörgenTate, Shin-ichiIshibashi, KenichiTateishi, KensukeIshida, TakashiTathireddy, PrashantIshii, TetsuroTatsuke, TsuneyukiIshii, YumikoTatullo, marcoIshikawa, YasuyukiTaub, MaryIshimaru, NaozumiTaube, JoeIshimoto, TakatsuguTaura, KojiroIshizaki, TakumaTavanti, FrancescoIslam, Md SorifulTavares, AnthonyIsman, Murray B.Tavío, María Del MarIsoda, KatsuhiroTavladoraki, ParaskeviIsola, GaetanoTavori, HagaiIsozaki, TakeoTawfik, Sherif AbdulkaderItahana, KojiTaylor, Erika A.Itami, TashiakiTaylor, KathrynIto, NaokiTchorbanov, AndreyIto, ShosukeTe Riele, HeinIto, TakuhiroTe Velthuis, AartjanIto, ToshiroTebani, AbdellahIto, YasuhiroTedeschi, RosemarieIto, YujiTedesco, IdoloItoh, TohruTedesco, SerenaItokazu, YutakaTee, Andrew R.Itou, JunjiTeeri, TeemuIvancic Santek, MirelaTeige, MarkusIvanov, Alexander G.Teixeira Tomaz, CândidaIvanov, Alexander V.Teixeira, Ana LuísaIvanov, Alexander VladimirovichTejada, SilviaIvanov, AndreyTelser, JoshuaIvanova, Magdalena I.Temel, AslihanIwai, AtsushiTemeyer, Kevin B.Iwaizumi, Masakazu G.Tempera, GiannaIwamoto, HiroyukiTempera, ItaloIwamoto, ShotaroTempfer, HerbertIwata, JunichiTencerova, MichaelaIwona, NiedzielskaTenhaken, RaimundIzasa, HisashiTennessen, JasonIzawa, TakashiTeraishi, MasayoshiIzawa, TakeshiTERAMOTO, HidetoshiIzuhara, KenjiTeramoto, YoshikuniJaaro-Peled, HannaTerpitz, UlrichJablonska, EwaTerracciano, DanielaJablonska, KarolinaTerreri, SaraJackson, ChristopherTerriente, JavierJacob, FrancisTerry, Randall G.Jacobs, DelphineTerzaghi, WilliamJacobs, Elizabeth R.Terzaghi, William BryanJacomin, Anne-ClaireTerzi, ValeriaJacques, DanielleTeschendorff, Andrew E. Jacquin, LisaTeschke, RolfJadavji, Nafisa M.Tessarz, PeterJadhav, Gopal P.Testa, UgoJaeschke, HartmutTestai, Fernando D.Jaffar, ZeinaTeter, KenJagannathan, VidhyaTetreau, GuillaumeJain, AshishTetz, GeorgeJain, VaibhavTevosian, Sergei G.Jaing, Tang HerTeymourian, HazhirJaiswal, Amit K.Tezil, TugsanJaiswal, Ashvin R.Thackray, AlanaJaiswal, J. K.Thakor, NehalJaligot, EstelleThakurela, SudhirJames, WilliamThaler, RomanJamwal, RohitashThalhammer, GregorJanczar, SzymonThammina, ChandraJanczarek, MonicaThangaraj, AnnaduraiJanda, ElzbietaTheile, DirkJanda, TiborThelin, EricJanetka, JamesThiel, TeresaJanevska, SlavicaThijs, SofieJang, JinahThilmony, RogerJang, Sung-WukThiriet, MarcJanga, MadhusudhanaThomas, PeterJänig, WilfridThomas, T. J.Janikowska, GrażynaThomas, WayneJaniuk, IzabelaThomine, SébastienJanowiak, Blythe E.Thompson, JeffreyJanowski, MiroslawThompson, Karl ManleyJansen, GerritThompson, KimJansen, RickThomson, JenniferJansky, ShelleyThorn, PeterJanuchowski, RadoslawThornburg, NatalieJanusz, GrzegorzThornton-Kurth, KaraJaradat, AbdullahThorpe, ChavaunneJaradat, NidalThrasyvoulou, AndreasJarajapu, YagnaThrivikraman, GreeshmaJara-Palacios, María JoséThummel, RyanJardin, IsaacThyparambil, AbyJardine, KolbyTian, BinJarret, Robert L.Tian, JiangJarstfer, Michael BruceTian, LiJarząb, BarbaraTian, XiaoyuJat, Parmjit S.Tian, ZhongminJaumot, JoaquimTiano, LucaJaurand, Marie-ClaudeTicozzi, NicolaJayant, Rahul DevTiera, MarcioJayaram, LataTietjen, IanJayasinghe, SuwanTiffen, Jessamy C.Jazirehi, Ali R.Tigyi, Gábor J.Jelena, KrsticTikhonov, Denis B.Jellinger, Kurt A.Tikhonov, VladimirJena, Prasant KumarTilbrook, AlanJeng, Jiiang-HueiTilley, MichaelJenkins, PaulTimani, KhalidJenkins, Samir V.Timofeev, Vladimir I.Jensen, Eric D.Timoshenko, Alexander V.Jensen, Lars HenrikTiriveedhi, VenkataswarupJeon, Byeong HwaTischler, DirkJeong, DaewonTiwari, PoojaJeong, Hyun YoungTiwari, Rakesh K.Jeong, JinyoungTiwari, VijayJeong, Keun-YeongToca-Herrera, JoseJeong, Seon-YongTocchetti, Carlo GabrieleJeppe, EmmersenToden, ShusukeJerebtsova, MarinaTodi, SokolJeremy, Teoh Yuen ChunToh, Ban-HockJerome, De RuyckToh, Wei SeongJeschek, MarkusToietta, GabrieleJeschke, Marc G.Toillon, Robert-AlainJeschke, UdoToiu, AncaJessen, NielsTokas, TheodorosJetten, AntonToldrá, FidelJetybayev, Ilyas YerkinovichTolkatchev, DmitriJeung, Eui-BaeTolosa, EzequielJeynes, CharlieTomar, DhanendraJeziskova, IvanaTomasetti, CarmineJha, AmitTomasetti, MarcoJha, MithileshTomassetti, AntonellaJha, SudhakarTomasson, MichaelJi, PengTomaszewska, EwaJi, TianjiaoTomatsu, ShunjiJI, YuelongTombesi, SergioJiang, Chen ChenTomczykowa, MonikaJiang, JianxiongTominaga, MotokiJiang, Qiu-XingTommasini, Steven M.Jiang, Shu-YeTomoaki, SakamotoJiang, SizunTomuleasa, CiprianJiang, TaoTong, QiangJiang, XinyinToniolo, Antonio Q.Jiang, XiqianToorie, AnikaJiao, ChenTooze, ReubenJiao, YinpingTop, DenizJimbo, MitsuruTorigoe, KanjiroJiménez-Alemán, GuillermoTornesello, MariaJimenez-Rondan, FelixTorras, JoanJimi, EijiroTorre, Maria LuisaJin, Dong-HoonTorrente, AngeloJin, FanTorres, Adrian GabrielJin, JiefuTorres, MagdalenaJin, XinTorres-Reveron, AnnelynJin, Young WooTorrieri, ElenaJindra, MarekTortora, PaoloJindrich, JindrichTorzewski, MichaelJinping, ZhaoTósaki, ÁrpádJirkovská, AnnaTosato, GiovannaJirkovsky, EduardTosh, SusanJo, Young SukTotah, RheemJoão, BenevidesToulany, MahmoudJob, DominiqueTouma, ChadiJochum, ChristophTovoli, FrancescoJohn, RohanTrabace, LuigiaJohnen, GeorgTrabucchi, MicheleJohnson, Andrew D.Traini, ToninoJohnson, Daniel E.Tran, FabienJohnson, ErikTran, KimJohnson, Jay E.Tranulis, MichaelJohnson, KimTravaglione, SaraJohnson, Lance A.Travers, Jeffrey BryantJohnson, Thomas V.Trdan, StanislavJohnsson, MartinTrejo, JoannJohnston, CarolTrenti, AnnalisaJohnstone, ScottTrentini, AlessandroJohnstone, Scott R.Tresguerres, JesusJoles, JaapTresguerres, JesúsJonassen, ChristineTretyakova, NataliaJones, AleckTrevisan, AndreaJones, MalcolmTriaca, VivianaJones, Mark G.Tribulova, NarcisaJones, MerielTricarico, DomenicoJonušienė, VioletaTrifonov, VladimirJoo, Kyeung MinTrigatti, BernardoJoo, YuyoungTrillas, M. IsabelJordi, GomezTrilling, MirkoJørgensen, Anders PalmstrømTrimboli, PierpaoloJørgensen, Hans Jørgen LyngsTrimigno, AlessiaJørgensen, KirstenTrinchera, MarcoJosef, KapfhammerTrincone, AntonioJoseph, SusanTrinick, JohnJoshi, BharatTripathi, DiwakerJoubert, OlivierTripathi, ManishJouk, Pierre SimonTripathi, ManojJoung, Hyou-ArmTrivedi, DarshanJovanović, AleksandarTrivedi, PankajJovanović, DraganaTrobaugh, DerekJovanovic-Santa, SuzanaTroiano, GiuseppeJóźwicki, WojciechTroisi, JacopoJuarranz, ÁngelesTrojanowicz, BoguszJubeli, EmileTrollope, AlexandraJuhas, StefanTronel, ClaireJulve, JosepTroppmair, JakobJung, Chol-HeeTrubiani, OrianaJung, Ki-HongTrujillo-Rodríguez, María JoséJung, KlausTruong, Vi KhanhJung, Sung-ChulTruve, ErkkiJurczyk, BarbaraTryndyak, VolodymyrJurenka, RussellTrzeciak, Anna M.Jurikova, TundeTsafantakis, NikolaosJuriloff, Diana M.Tsai, Ang-ChenJust, NathalieTsai, Inn-HoJust, SteffenTsai, Kun-LingK Song, MoonTsai, Shih-changKabała-Dzik, AgataTsai, Shih-JenKabbage, MehdiTsai, Shu-HuaiKabil, OmerTsai, Yuan-ChinKačarević, Željka PerićTsapogas, PanagiotisKachamakova-Trojanowska, NeliTsatsakis, Aristides M.Kaczmarek, BeataTsavkelova, Elena A.Kaczorek, EwaTse, William K.F.Kadam, SuhasTse-Dinh, Yuk-ChingKaddoumi, AmalTseng, Te-Ming PaulKaddour, HusseinTseng, Yi-TangKadimisetty, KarteekTsiotra, Panayoula C.Kadokawa, Jun-ichiTsirigotis, PanagiotisKagota, SatomiTsitsilonis, Ourania E.Kai, MihokoTsoulfas, GeorgeKaina, BerndTsoyi, KonstantinKajava, AndreyTsubaki, KazunoriKajiya, HiroshiTsuboi, MasahitoKajiya, MikihitoTsuchida, SachioKakehashi, AnnaTsuchiya, HiroyukiKakinen, AleksandrTsuchiya, YuichiKakumanu, AkshayTsuda, TakeshiKalani, KomalTsuji, Atsushi B.Kalatzis, VasilikiTsuji, GakuKalayda, GannaTsujimoto, HisashiKalber, TammyTsukahara, TamotsuKaldis, PhilippTsukahara, ToshifumiKale, AbhijitTsutsui, MakusuKalhapure, RahulTsutsui, ShigeyukiKaliniewicz, ZdzisławTsytsarev, VassiliyKallergi, GalateaTu, ThomasKallikourdis, MarinosTuanyok, ApichaiKalogirou, CharisTuccinardi, TizianoKalthoff, HolgerTuchscherr, LorenaKaluderovic, GoranTucker, StevenKam, Yiu WingTueros, ItziarKamagata, KiyotoTuite, MickKamal, Abu Hena MostafaTulloh, RobertKamath, DivyaTunaru, SorinKamato, DanielleTuncel, AytugKaminaka, HironoriTuomikoski, PauliinaKaminski, RafalTuorto, FrancescaKamitori, ShigehiroTurano, PaolaKamolz, LarsTuriel, MaurizioKanakkanthara, ArunTurkina, Maria V.Kanakry, Christopher G.Turnbull, Matthew W.Kanasak, KeizoTurner, JonathanKanazawa, MasatoTurner, MarkKanda, TeruTuroverov, KonstantinKandimalla, RajuTurowski, PatricKanekiyo, TakahisaTurrini, EleonoraKaneko, GenTurroni, SilviaKanematsu, HideyukiTuttolomondo, AntoninoKang, ChenTuvdendorj, DemidmaaKang, Chul HeeTuveng, Tina RiseKang, CongBaoTuyaerts, SandraKang, Hee Y.Tyagi, RahulKang, Sang SunTyrka, MirosławKang, SinyoungTyunin, Alexey P.Kang, Tae-HongTzin, VeredKang, WeiTzinia, Athina K.Kang, Young-HeeUberti, FrancescaKannan, RamUchida, ShizukaKanojia, DeepakUdumula, Venkata ReddyKant, SuryaUegaki, KoichiKanzaki, HiroyukiUeno, Shu-ichiKao, Shu-HueiUfnal, MarcinKaplan, ShaunaUhl, EberhardKaplinsky, Nicholas J.Uhrikova, DanielaKappachery, SajeeshUlasov, IlyaKaput, JimUlf, KahlertKarabagias, IoannisUlivieri, CristinaKarafiloglou, PadeleimonUlman, VladimírKaragöz, ElifUlrich, CraigKarakasidis, T. E.Umar, Akhilesh KKaraki, Shin-ichiroUmehara, MikihisaKarakoula, KatherineUmerska, AnitaKarakülah, GökhanUnchwaniwala, NuruddinKaran M, ShahUnderwood, Mark D.Kararigas, GeorgiosUngefroren, HendrikKarasiński, JanuszUpadhyay, Rakesh K.Kardos, Józsefur Rehman, IhteshamKärenlampi, SirpaUrano, DaisukeKarganova, Galina G.Uray, IvanKari, LeifUrban, JillKarkanis, AnestisUrban, PhilippeKarlic, HeidrunUrbanczyk-Lipkowska, ZofiaKarlsson, ErikUrbanek, TomaszKarmakar, MausitaUrbanelli, LorenaKarmakar, ParthaUrbano, Ana MargaridaKarmazyn, MorrisÚrbez-Torres, José RamónKarpiński, Tomasz M.Usmani, AbulKasahara, Ryushiro D.Üstün, SuayibKaschula, Catherine H.Usui, SoichiroKasetti, RameshUusitupa, MattiKashiwagi, ShinichiroUversky, Vladimir N.Kashtalap, VasilyUziel, OritKashuba, ElenaVaahtera, LauriKasinath, Balakuntalam S.Václavíková, RadkaKasoju, NareshVadivel, KanagasabaiKasparkova, JanaVadlapudi, Aswani DuttKast, Richard EricVafiadaki, ElizabethKasten-Jolly, JaneVågesjö, EvelinaKatada, KazuhiroVaghefi, EhsanKataoka, NaoyukiVago, RiccardoKatarivas Levy, GalitVaillancourt, CathyKatayama, KentaroVaiman, DanielKateřina, SchwarzerováVainio, SeppoKathuria, HimanshuVairetti, MariapiaKato, J.Vaisar, TomášKato, KoichiVajro, PietroKato, SoValabrega, GiorgioKato, Takamitsu A.Valdes-Lopez, OswaldoKato, YasumasaVale, Filipa F.Katsantonis, DimitriosValentine, Rudy J.Kaul, MarcusValentová, KateřinaKaunas, Roland R.Valentovic, MonicaKauppinen, AnuValenzuela, C. FernandoKaur, AmanpreetValenzuela, StellaKaur, JasmineValero, Marta SofíaKavita, KumariVali, PayamKawabata, KohsukeValko, MarianKawabata, KyuichiValkov, VladimirKawabe, AkiraValle, AdamoKawaguchi, Shin-ichiVallée, AlexandreKawai, TakayukiVallera, DanielKawakami, HiroshiValles, Soraya L.Kawakami, SusumuValletta, AlessioKawanami, DaijiValverde, Ángela M.Kawano, KouichiroVamanu, EmanuelKawasaki, HidekiVamecq, JosephKawase, TomoyukiVan Aken, OlivierKawata, KazumiVan Beuningen, Henk M.Kawauchi, TakeshiVan Cruchten, StevenKay, GemmaVan Cutsem, PierreKayano, Shin-ichiVan Dam, NicoleKayser, KlausVan De Poel, BramKazama, TomohikoVan De Ven, RienekeKazeto, YukinoriVan Deenen, NicoleKazmi, FarazVan Den Bergh, HubertKe, Liang-YinVan Der Does, Anne M. Ke, YouqiangVan Der Giezen, MarkKeely, Simonvan der Kamp, Jan WillemKefalov, VladimirVan Der Kuyl, AntoinetteKeil, KimberlyVan Der Linde, KarinaKeller, AndreasVan Der Spoel, Aarnoud C.Kelley, DarshanVan Der Veer, Eric P.Kelley, EricVan Der Vorst, EmielKello, MartinVan Dyke, Michael W.Kelly, Michy P.van Eeden, StephanKemp, Bruce E.Van Esse, WilmaKemp, Michael G.Van Eynde, AleydeKempińska-Podhorodecka, Agnieszka D.Van Grevenynghe, JulienKenchanmane Raju, Sunil KumarVan Griensven, Martijn Kendig, MichaelVan Horn, WadeKenealey, JasonVan Loo, Hanna M.Kenichi, SakuraiVan Meeteren, Laurens A.Keniya, Mikhail V.Van Noorden, CornelisKennedy, DavidVan Raamsdonk, Jeremy M.Kenny, Hilary A.Van Rijn, Richard M.Kerwin, Rachel E.Van Roosbroeck, KatrienKerz, ThomasVan Royen, NielsKessel, DavidVan Spil, Erwin (W.E.)Kevadiya, BhaveshVan Tine, Brian AndrewKhairullina, VeronikaVan Vliet, SandraKhalil, ChristianVan Zandwijk, NicoKhalil, ZeinabVan Haute, LindseyKhan, Atif AliVance, David E.Khan, Mohd ParvezVandeputte, DorisKhan, MohsinVandooren, JenniferKhan, MuhammadVaňhara, PetrKhapli, SachinVankayala, RavirajKharbanda, Kusum K.Vanková, RadomiraKharissov, Boris IldusovichVanneste, SteffenKharkar, PrathameshVanni, RobertaKhasawneh, FadiVannini, IvanKhatodia, SurenderVáradi, GyörgyiKhatri, BhuwanVarallyay, EvaKhatri, KshitijVarando, GherardoKhattab, MuhammadVaranini, ZenoKhazaei, HamidVarela-Ramirez, ArmandoKhlebtsov, Nikolai G.Varese, Giovanna CristinaKhoda, BashirVarghese Chacko, JenuKhodanovich, MarinaVarikuti, SanjayKhoshmanesh, KhashayarVarotsos, CostasKhotimchenko, Maxim Y.Varotto, SerenaKhuder, Sadik A.Varricchio, EttoreKhudyakov, JaneVarshavsky, Alexander J.Khurshid, ZohaibVasaikar, SuhasKi, Sung HwanVashist, Sandeep K.Kiaei, MahmoudVasilevsky, NicoleKianifariz, MahnazVasin, Mikhail V.Kibriya, MuhammedVassetzky, YegorKiefer, FriedemannVasudevan, Ananda Ayyappan JaguvaKiełbowicz-Matuk, AgnieszkaVathipadiekal, VinodKielbus, AndrzejVaudry, HubertKiełczykowska, MałgorzataVaughan, Roger A.Kieliszek, M.Vautier, SimonKieliszek, MarekVázquez-Marrufo, GerardoKielland-Brandt, MortenVecchione, CarmineKienesberger, PetraVecchione, RaffaeleKietzmann, ThomasVechetti, IvanKikis, Elise A.Vecoli, CeciliaKikowska, MałgorzataVeenman, LeoKikuta, ShingoVeenstra, Richard D.Kilari, SreenivasuluVeeramani, SureshKilinc, DevrimVeeraraghavan, RengasayeeKiliszek, AgnieszkaVega Gutierrez, SarathKilpatrick, LauraVega, Francisco M.Kim, AeRangVega, Gladys MínguezKim, BhumsooVégh, Attila GergelyKim, Byung-SooVeiga-Lopez, AlmudenaKim, Chang KilVeikko, LinkoKim, Chang WookVelasco, GuillermoKim, Cheorl-HoVeldkamp, ChristopherKim, ChoelVeleeparambil, ManojKim, Dong JoonVélez-Cruz, RenierKim, DonginVelitchkova, MayaKim, Dong-SeonVelivelli, SivaKim, DongukVellante, FedericaKim, HangunVemula, HarikaKim, HoonVenditti, AlessandroKim, Hoy-TaekVennerstrom, JonathanKim, Hun SikVentoso, IvánKim, Hye OneVenuti, AldoKim, HyesunVenza, MarioKim, Hyeung-RakVerdeguer, FranciscoKim, HyounjuVerdoucq, LionelKim, Hyun AhVergara, DanieleKim, Hyun JoonVerhage, LeonieKim, Hyun SoonVerhoeven, AdrieKim, Hyung SikVerma, Navin KumarKim, Hyung-JoonVermaas, Joshua V.Kim, In-JungVernetti, Lawrence A.Kim, InKyeomVeronese, MattiaKim, J. JulieVeronesi, FabioKim, Jeong-RaeVéronique, Demers-MathieuKim, Jin-BomVéronique, SantoniKim, Jin-WookVerrey, FrançoisKim, JoungmokVerron, EliseKim, Ki HyunVersacci, PaoloKim, Kyoung SooVersino, ElisabettaKim, KyounghyunVerstegen, Monique M. A.Kim, Kyung-MinVerstraeten, IngeKim, Kyung-suVersura, PieraKim, Moon KiVervaet, BenjaminKim, Nam DeukVeskoukis, Aristidis S.Kim, Nam-JungVesper, StephenKim, Sang-WeVessey, KirstanKim, SeonghoVetere, AmedeoKim, SunggilVeyron-Churlet, RomainKim, Sung-HoonVicente, ClaudiaKim, Sung-KunVicente, JoaoKim, Tae IlVickers, MarkKim, Tae-HyungVictoni, TatianaKim, TaewanVictor, BrunoKim, WeonVidalain, Pierre-OlivierKim, WoojinVidic, JasminaKim, YangmeeVieira, Maria Lúcia CarneiroKim, Yong-miViennois, EmilieKim, YoungsooViganò, PaolaKim, Yun KyungViggiano, DavideKim, Yun-baeVignes, MichelKimber-Trojnar, ŻanetaVijayakumar, SarathKimbrel, ErinVijayavenkataraman, SanjairajKimler, BruceVilla, ChiaraKimura, KeiVilla, StefaniaKimura, MotokoVilla-Bellosta, RicardoKimura, SeisukeVillalobos, CarlosKindy, MarkVillalta, PeterKing-Lyons, Natalie D.Villarino, GonzaloKirberger, MichaelVillar-Lozano, DiegoKirillova, AlinaVillar-Piqué, AnnaKirsch, ThorstenVillasante, AranzazuKirschnek, SusanneVillers, AgnèsKirton, Stewart B.Vinardell, José M.Kishaba, TomooVinardell, MariaKishi, TakuyaViñas, MiguelKishikawa, NaoyaVincenzo, De LeoKishimoto, YoshimiVinciguerra, ManlioKiskova, TereziaVinken, MathieuKiss, AnnaVinkler, MichalKiss, JohnVinnedge, Lisa PrivetteKiss, Robert S.Virag, LaszloKitagawa, SeiichiVisconti, Richard P.Kitamura, NaokiVisioli, FrancescoKitamura, TakanoriVistoli, GiulioKitaoka, SatoshiVita, RobertoKitatani, KazuyukiVitale, AlessandroKitchen, PhilipVitale, MarilenaKitson, Matthew T.Vítámvás, PavelKittaka, AtsushiVitetta, LuisKiyan, YuliaVitry, PaulineKjøbsted, RasmusVivo, MariaKlaassen, Curtis D.Vizoso, Francisco J.Klajner-Maculewicz, BarbaraVlachos, IoannisKlajnert-Maculewicz, BarbaraVladimirov, VladimirKlampfer, LidijaVliagoftis, HarissiosKlapdor, RüdigerVo, MinhKlar, AgnesVoelckel, ClaudiaKlassen, RolandVoelkers, MirkoKlaus, SusanneVoellger, BenjaminKleczkowski, Leszek A.Vogelaar, Christina FranciscaKlein, AxelVoiniciuc, CătălinKlein, Janet D.Vojta, LeaKleine, TatjanaVolarevic, VladislavKlementieva, OxanaVolk, DavidKleppe, LeneVolonté, CinziaKlettner, AlexaVon Delft, FrankKlimek, KatarzynaVon Eckardstein, ArnoldKlokov, Dmitry Y.Von Knethen, AndreasKlontzas, MichaelVon Kobbe, CayetanoKlose, HolgerVon Zglinicki, ThomasKlose, JohannesVona, BarbaraKlotz, JamesVora, MehulKlugbauer, NorbertVoss, UteKlusa, VijaVoutsadakis, Ioannis A.Kluza, JéromeVozza, IoleKlychnikov, OlegVrentas, Catherine E.Knap, NarcyzVriend, JerryKnecht, HansVrontaki, EleniKnepper, JaniceVukojevic, VladanaKnight, DavidVuts, JozsefKnight, Jason S.Vyazovkin, SergeyKnight, VijayaVymetalkova, VeronikaKnipper, Johanna A.Vyoral, DanielKnoch, EvaWabiko, HiroetsuKnoell, DarenWachten, DagmarKnoell, RalphWada, JunKnott, RachelWada, Ken-IchiKnox, PaulWada, RyuichiKo, Jane L.Waddell, JaylynKo, Jiunn- LiangWade, RebeccaKobayashi, JunWadehra, MadhuriKobayashi, Natsuko I.Wagh, KshitijKobayashi, SatoruWagner, AnikaKobayashi, YayoiWagner, GabrieleKobayashi, YoshiroWagner, LarsKobayashi, YutaroWagner, Lars M.Kobeissy, Firas H.Wahli, WalterKöbel, MartinWakao, MasahiroKoch, AlexanderWakimoto, HiroakiKoch, StefanWako, HiroshiKoch, WojciechWakshlag, JosephKocot, JoannaWalczak, KatarzynaKodama, HiroakiWalentowicz-Sadlecka, MalgorzataKoepke, TysonWaligorski, PiotrKoga, Jun-ichiroWalker, Chandler L.Kogashiwa, YasunaoWalker, HeatherKoh, Cho YeowWallace, NicholasKoh, Gar YeeWaller, DeborahKoh, Ho-JinWaller, RachelKöhl, GudrunWall-Medrano, AbrahamKoibuchi, NoriyukiWalsh, Laurence J.Koike, KenzoWalters, GilesKoivisto, AriWalton, Gemma EmilyKoizume, ShiroWan, EdwinKojima, ChojiroWan, Lam YimKojima, MasamiWan, ShibiaoKojima, ShihokoWan, XuehuaKojima-Yuasa, AkikoWanders, DesireeKok, DieuwertjeWang, Bang-AnKok, WouterWang, BoKokabu, ShoichiroWang, Chin-KunKolbe, LudgerWang, FuyiKolesárová, AdrianaWang, GuliangKolettas, EvangelosWang, GuozhengKoller, GabrieleWang, HsiuyingKolluri, Siva KumarWang, HuaiminKolluru, GopiWang, Hui-Min DavidKolmas, JoannaWang, JenghanKolodych, SergiiWang, JingKolosov, DennisWang, JiouKolosov, PeterWang, JunKolukisaoglu, ÜnerWang, KaiKomatsu, KenWang, LingKomine, MayumiWang, Lisa LKomis, GeorgeWang, LizhuKommineni, VallyWang, PengchengKommireddy, VasuWang, Peng-HuiKonar, ArkaprabhaWang, PiwenKondo, HidehiroWang, QiangKondo, TatsuyaWang, QinKondratieff, BorisWang, QunKoneru, BhuvaneswariWang, RanranKones, RichardWang, Raymond Y.Kong, YimengWang, RichardKönig, DanielWang, RuiKoninckx, Philippe R.Wang, San-LangKonishi, MasaakiWang, ShengKonno, HiroyukiWang, Shih-WeiKonopka-Postupolska, DorotaWang, ShyiwuKonstandin, MathiasWang, Tsung-JenKonstantinov, Spiro M.Wang, Wen-DerKontny, UdoWang, WenqiKontogiannatos, DimitriosWang, WentianKontoravdi, CleoWang, XiaowanKoo, HeebeomWang, XinkunKopecki, ZlatkoWang, XiupengKopecký, Vladimírwang, xuewenKopel, PavelWang, YifeiKoppe, LaetitiaWang, Yi-FenKorać, PetraWang, YingKorasick, David A.Wang, Yun-MingKorenjak, MichaelWang, ZheKorfei, MartinaWang, ZhihanKorkaya, HasanWard, DouglasKorkina, Ludmila G.Ward, Sara JaneKornelyuk, AlexanderWardill, Hannah R.Korou, Laskarina-MariaWarzecha, TomaszKorsching, EberhardWarzecha, ZygmuntKorte, CassandraWase, NishikantKoschella, AndreasWasinger, Valerie C.Kosmas, Constantine E.Wasternack, ClausKosova, KlaraWasylishen, AmandaKostakis, IoannisWatanabe, MasakatsuKosters, AstridWatanabe, MasatoshiKostyuk, Vladimir A.Watanabe, MasayukiKotsikorou, EvangeliaWatanabe, MikiKotsinas, AthanassiosWaterhouse, MiguelKotta-Loizou, IolyWaters, MichaelKotula-Balak, MalgorzataWatrelot, Aude AnnieKotyla, Przemyslaw J.Watson, LaurenKoubassova, NataliaWatson, WilliamKoul, HariWattanapitayakul, Suvara K.Koulen, PeterWayel, JassemKoupenova, MilkaWdowiak, KamilKourkoumelis, NikolaosWeaver, JolantaKousik, Chandrasekar S.Weber, DanielKoutelidakis, Antonios E.Weber, David S.Kovac, StjepanaWeber, FranzKovacs, RichardWeber, Georg F.Koval, AlexeyWeber, JohnKoval, MichaelWeber, LynnKovbasnjuk, OlgaWebster, Marie R.Kovermann, MichaelWei, ChangyongKovinich, NikWei, YonglongKowalczuk, MarekWei, YufengKowalska, KatarzynaWeigand, Julia E.Kowtharapu, Bhavani S.Weigert, AndreasKoya, DaisukeWeighardt, HeikeKoyama, RyutaWeigmann, BennoKoyama, YuWeil, Michael M.Kozaki, AkikoWeiskopf, DanielaKoziak, KatarzynaWeiss, JuliaKozieł, EdmundWellington, CherylKozikowski, AlanWellmann, SvenKozlov, KonstantinWells, James W.Kozlova, ElenaWells, Richard A.Kozlowski, Michael R.Wells, TimKraemer, Brian C.Welsch, RalfKraft, RobertWelsh, C. JaneKraic, JanWelsh, MichaelKrajewska-Włodarczyk, MagdalenaWemheuer, FranziskaKramer, PhillipWen, Chiu-MinKramnik, IgorWen, ChunyiKranz, StefanWen, JianjunKrasowska, AnnaWende, WolfgangKrause, GünterWendell, Stacy GelhausKrebs, JoachimWeng, Mao-LunKren, VladimirWeng, XiaoyuKrenács, TiborWenisch, SabineKrenn, Veit T.Wenkel, StephanKrenz, BjörnWennmalm, StefanKreyling, WolfgangWerley, Christopher A.Krick, StefanieWesolowski, RobertKriebardis, Anastasios G.Wessells, RobertKrishnan, NatrajWessinger, CarolynKrishnan, SanalkumarWest, Alison C.Kristensen, Lasse SommerWest, Lori J.Kristensen, MieWest, TimKrogan, Naden T.Weyer, AndyKról, EwelinaWhelan, Rebecca J.Kroncke, Brett M.White, ElizabethKropski, Jonathan A.White, PeterKrycer, JamesWhitfield, PhilKschischo, MaikWhitford, Paul CharlesKu, Kang-MoWibowo, DavidKu, Seung-YupWickner, Reed B.Kuan, Yu-HsiangWidmer, Hans RudolfKubaczka, CarolineWie, Myung-BokKubala, SzymonWieckiewicz, MieszkoKubatka, PeterWieczorek, EdytaKubíček, VojtěchWieczorek, Rafal M.Kubina, RobertWiemer, Erik A.C.Kubinova, SarkaWierichs, RichardKubiński, KonradWieser, RotraudKubista, HelmutWietrzyk, JoannaKubo, AtsushiWiggins, GregKubo, NakaoWiggs, MichaelKubo, TakanoriWigle, JeffreyKubo, ToshioWigley, DaleKubota, ToshiakiWijeyeskere, SanjeevaKuca, KamilWildemann, BrittKuçi, SelimWilker, JonathanKucinska-Lipka, JustynaWilkinson, J. M.Kucuk, IsrafilWilkinson, Mark D.Kucukkal, TugbaWillberg-Keyriläinen, PiaKudłak, BłażejWillert, KarlKudo, YasuseiWillette, Auriel A.Kudryasheva, Nadezhda S.Williams, Christopher S.Kudryashova, Tatiana V.Williams, StephenKuebler, DanWilliams, Steven E.Kuehl, MichaelWillis, RohanKues, Wilfried A.Willison, HughKuhla, AngelaWilson, CliveKuhlmann, MarkusWilson, Eleanor M.P.Kuhn, AndreasWilson, TedKühnlein, RonaldWinchester, Laura M.Kujan, OmarWinek, KatarzynaKujawska, MałgorzataWinkler, JohannesKukimoto, IwaoWinship, AmyKukley, MariaWińska, KatarzynaKukula-Koch, WirginiaWinuthayanon, WipaweeKulawiak, BoguszWischhusen, JoergKulbacka, JulitaWischik, ClaudeKulma, AnnaWiśniewski, MarekKulus, DariuszWitczak, CarolKumamoto, EiichiWiteska, MałgorzataKumar, AmitWitowski, Jan SylwesterKumar, BinodWitt, MartinKumar, DevenderWitte, HartmutKumar, GauravWitten, Paul EckhardKumar, GokhleshWittstock, UteKumar, ManojWlaz, PiotrKumar, ManuWłoga, DorotaKumar, NarenderWodrich, HaraldKumar, PradeepWójkowska, Dagmara WeronikaKumar, RajivWojtas, BartoszKumar, SathishWojtyczka, RobertKumar, SujeetWojtyla, ŁukaszKumar, SunilWölfler, AlbertKumar, SushilWolińska, AgnieszkaKumar, UjendraWolley, MartinKumar, VasanthWon, Kyu ChangKumari, AshaWon, Kyung JongKumasawa, KeiichiWong, AlanKumi-Diaka, JamesWong, Boon-SengKundu, AishwaryaWong, Chi-MingKundu, ParagWong, Ching-OnKunej, TanjaWong, Chung F.Kung, Yu-ChunWong, ConnieKunioka, MasaoWong, Ka-ChunKunz, MeikWoo, Se JoonKunz, WolframWoo, So-YounKuo, Cheng-ChinWoodhead, Jeffrey L.Kuo, LihWoodman, TimKuo, Ping-ChungWoolbright, Benjamin L.Kuo, Po-LinWorch, RemigiuszKuo, Tzong-FuWorkman, VictoriaKupai, KrisztinaWorrell, RogerKupčinskas, JuozasWoulfe, Kathleen C.Kupcova Skalnikova, HelenaWoźniak, ŁukaszKuppusamy, ManiselvanWright, CatherineKurakula, KondababuWrzaczek, MichaelKurapati, RajendraWrześniok, DorotaKurczynska, EwaWu, ErxiKurdowska, Anna K.Wu, FenKurgan, LukaszWu, GuangxiKurien, Biji TheyilamannilWu, HannahKuroda, MasashiWu, Kuen-phonKurokawa, TetsujiWu, ShuKurreck, JensWu, SiKurrikoff, KaidoWu, Ting-FengKurschus, FlorianWu, Tzong-YuanKurth, InaWu, YanKuryk, LukaszWu, YanyuanKurylowicz, AlinaWu, Yi-YingKuryłowicz, AlinaWu, Yuh-LinKurz, Jacqueline LaRoseWunderlich, FrankKurzyńska-Kokorniak, AnnaWürth, RobertoKus, PiotrWybouw, NickyKus-Liśkiewicz, MałgorzataWynes, Murry W.Kusmartsev, SergeiWyns, ChristineKusters, IljaWyrobek, Andrew J.Kusuma, GinaWystrychowski, GrzegorzKuter, KatarzynaXavier, Nuno ManuelKuzmin, IvanXia, TianKuznetsova, Irina M.Xiang, YuKuźnicki, JacekXiao, LiKuźnik, NikodemXiao, YuKuzuya, AkinoriXiaoli, AlusKwak, Brenda R.Xie, HongKwakowsky, AndreaXie, RanKweon, Oh-KyeongXie, WankunKwiatkowska, KatarzynaXie, Zhong-RuKwiecien, Jacek M.Xin, DongyueKwok, Seung-KiXin, RanKwon, Jae-SungXing, GengmeiKwon, Kyoung JaXing, JunjiKwon, Tae-HwanXiong, YeKwon, Tae-YubXiong, ZhaohuiKwon, Young M.Xu, BoKwong, JosephXu, FengKyle, StuartXu, HuaxiKyoko, OkaXu, JingLa Barbera, GiorgiaXu, JinsongLa Ferla, BarbaraXu, JintaoLa Regina, GiuseppeXu, LuLa Rocca, Renato V.Xu, PengLa Rosa, Valentina LuciaXu, PengfeiLa Russa, DanieleXu, ShengshengLaBean, Thomas H.Xu, SuowenLabruna, GiuseppeXu, WayneLacal, Juan CarlosXu, WenyanLacham-Kaplan, OrlyXu, XinLachowiec, JenniferXu, YiLacis, GunarsXu, YungangLacomme, ChristopheXu, ZhijieLafarga, TomasXu, ZhipingLaforenza, UmbertoXuan, Tran DangLagali, PamelaYabe, KimikoLagana, Antonio SimoneYadav, Vikramaditya G.Lagger, SabineYadgarov, LenaLahiri, AmitYaegashi, HajimeLahiri, AmitabhaYager, DorneLahiri, TanayaYagi, AkiraLahooti, HooshangYago, ToruLai, Jui-YangYagüe, ErnestoLai, Tsung-HsuanYaish, Mahmoud W.Lai, Wen-Fu ThomasYakovlev, Igor A.Lai, YandongYalowich, Jack C.Lai, YingJuYamaguchi, JunpeiLaimins, LaimonisYamaji, ToshiyukiLakatos, BorisYamakuchi, MunekazuLakatos, LorantYamamoto, SuguruLam, Kwok HoYamamoto, YusukeLam, YuentingYamamura, AyaLamar, John MichaelYamamura, HisaoLamas, JesúsYamamura, ShoheiLamas, José RamónYamamura, SoichiroLamb, DavidYamanouchi, KeitaroLamba, DorianoYamasaki, MasahiroLambing, ChristopheYamashiro, SawakoLambrinidis, GeorgeYamashiro, TakashiLamichhane, PurushottamYamasu, KyoLamichhane, RajanYamauchi, TakahiroLamina, ClaudiaYamawaki, HideyukiLaMontagne, Michael G.Yamazaki, YuLampe, JedYan, DayunLan, LanYan, DongqingLan, PingYan, ShengminLan, Yuan-TzuYanda, MuraliLancelin, Jean-marcYang, Chih-TsungLandreh, MichaelYang, Chin-YingLandstrom, AndrewYang, Deng-HoLandström, MareneYang, GabsikLang, DiYang, GuangLange, EwaYang, Hui LinLangford, T. DianneYang, Jae WookLao, Ka UnYang, Jenq-LinLappano, RosamariaYang, Jen-TsungLaquintana, ValentinoYang, Jung-WuLaranjinha, João AntonioYang, Jun-YiLaranjo, MartaYang, Jye-ShaneLara-Sanchez, AgustinYang, KunLarner, AndrewYang, Lee-WeiLarraneta Landa, EnekoYang, LijiangLarsen, Anders ChristianYang, QiyuanLathia, Justin D.Yang, Rong-SenLatorre, Juan DavidYang, Seung HwanLattanzio, RossanoYang, Sherry X.Lau, KinYang, ShuyiLau, Kin-MangYang, Sze-Ming MildredLau-Cam, Cesar A.Yang, Ta-ILaudisi, FedericaYang, TaoLauf, Peter K.Yang, TuoLaurent, PatrickYang, WancaiLauritano, DorinaYang, Wei-hsiungLauro, ClotildeYang, XiaLauronen, JouniYang, YeLauschke, VolkerYang, YongkangLauth, MatthiasYang, Yueh-Hsun KevinLavandera, IvánYang, ZhihongLavogina, DarjaYann, AudicLayek, BuddhadevYannakopoulou, KonstantinaLayh-Schmitt, GerlindeYanni, SouzanLazado, C. C.Yano, TohruLazár, DušanYao, HiroshiLazar, ZsofiaYao, JiayiLe Hir, RozennYao, MasahiroLe Ouay, BenjaminYao, PengLe Trionnaire, GaëlYao, YanhuaLeach, Damien A.Yao, ZhiliLeak, Rehana K.Yarowsky, PaulLeanza, LuigiYasuda, KazukiLebar, MatthewYasuda, TakakoLebaron, RichardYasuhara, TakaoLeBlanc, RogerYates, ClaytonLebreton, SébastienYazlovitskaya, Eugenia M.Lebrun, VincentYe, AdamLecanda, FernandoYe, HaobinLechanteur, AnnaYe, YanqiLechel, AndreYeckel, Catherine WeikartLeclercq, GuyYeh, Chih-koLecuona, EmiliaYeh, Elizabeth S.Lecureur, ValérieYeh, Hsin-HungLedda, CaterinaYeh, Shu-LanLeddy, HollyYeh, Yin-TingLedo, AnaYehuda, ShoenfeldLee, Byeong JaeYeligar, SamanthaLee, Chang HoonYen, Feng-LinLee, Chang SupYen, Meng-ChiLee, ChangWooYeo, SynLee, Che-HsinYew, P. ReneeLee, Cheol KooYi, Ae-KyungLee, CheoljuYi, HaoweiLee, Chia-HungYi, Young-SuLee, Chia-HwaYilmaz, BahtiyarLee, Dae HoYin, ChengqianLee, GabsangYing, TiejinLee, GihyunYokohira, MasanaoLee, Hae-JuneYokoi, ShujiLee, HueiYokoi, TsuyoshiLee, HyoungseokYokoo, HirokiLee, Jae-HoYokota, ShinsoLee, JamesYonekura, ShinichiLee, JonghunYoneshiro, TakeshiLee, Joo HyoungYong, JeongsikLee, Jun HeeYoo, Ki-OugLee, KangwonYoo, SoonmoonLee, KeehoonYoo, Tag KeunLee, Li-TingYool, AndreaLee, MingeolYoon, Do-YoungLee, Myung KooYoon, HyonokLee, Sang-HanYoon, In-SooLee, SanghyeobYoon, Je-HyunLee, Seong-RyongYoon, Tae KiLee, Shin-DaYoshida, HiderouLee, Soo-HongYoshida, KatsunoriLee, Su-JunYoshida, ManabuLee, TaesupYoshida, TakahiroLee, Tong GeonYoshida, ToshinoriLee, Tzong-ShyuanYoshida, YamatoLee, Wing-KeeYoshihara, ToshinoriLee, Yoon KwangYoshino, HironoriLee, YunjongYoshioka, Ken-ichiLefebvre, HervéYoshitomi, HiroyukiLegay, ClaireYosten, GinaLegay, SylvainYou, HyunjuLeggio, LorenzoYou, SylvaineLegler, Daniel F.You, XiaonaLegros, ChristianYoung, ArabellaLehotsky, JanYoung, BryanLei, WeiYoung, Bryan G.Leijs, Marike M.Young, Jason C.Leiphrakpam, Premila D.Young, KayleneLeitão, AnaYu, BinLeitão, Jorge H.Yu, Chia-LiLeitinger, BirgitYu, Feiqiao BrianLeitner, AlexanderYu, GangLeiva-Brondo, MiguelYu, JianLekli, IstvánYu, RichardLembo, GiuseppeYu, Seung-YoungLemieux, KiTaniYu, VictorLemke, JohannesYu, Yan PingLemmens-Gruber, RosaYu, YangLenaghan, ScottYu, Yuan-HsiangLeng, RogerYuan, BoLeón, JosefaYuan, Hanna S.Leonard, MartinYuan, YaxiaLeonhardt, Ralf M.Yuan, ZhihongLeopold, Jane A.Yuba, EijiLephart, EdwinYue, PatrickLeporatti, StefanoYuen, John WmLerm, MariaYuen, Karen Wing YeeLeroux, ChristineYumoto, HiromichiLeroy, BaptisteYun, ChawonLeroy, JoYun, HongseokLescrinier, EvelineYun, HyungdonLesgards, Jean-FrançoisYurchenco, Peter D.Lesniak, WieslawaYushenova, IrinaLeso, VerusckaYuspa, Stuart H.Lestavel, SophieZabetakis, IoannisLesyk, Roman B.Zaccai, MicheleLeung, IvanhoeZaccheroni, NelsiLeung, Yuet-KinZacchia, MiriamLevesque, MitchellZacconi, Flavia C.Levi, AmnonZachou, KalliopiLevitskii, SergeyZafar, MuhammadLevkau, BodoZagidullin, NaufalLevy, StevenZahedi, KamyarLev-Yadun, SimchaZaibi, Mohamed SghaierLewin, Alfred S.Zaid, HilalLewinska, AnnaZaidi, Syed Shan-e-AliLewis, Jeffrey A.Zakhari, SamirLewsey, Mathew G.Zaki, HasanLeyva-Grado, VictorŽáková, LenkaLézot, FrédéricZakrocka, IzabelaLezza, Angela Maria SerenaZakrzewicz, DariuszLi, ChiZallot, Remi G.Li, ChunshunZambonelli, PaoloLi, DapengZambrano, SamuelLi, DeminŻamojć, KrzysztofLi, FuyangZamyatnin, Jr., Andrey A.Li, GuohuiZanelli, Cleslei FernandoLi, HuinanZang, LiqingLi, JinZani, Bianca MariaLi, Ka WanZaniboni, MassimilianoLi, KeshuaiZanin, LauraLi, LipingZannetti, AntonellaLi, MeichunZapata, CarlosLi, Mei-LingZaphiropoulos, PeterLi, NingZapico, Sara C.Li, Shengwen CalvinZarogoulidis, PaulLi, ShihaoZarra, IgnacioLi, TianhuZasloff, MichaelLi, WeijuanZaucke, FrankLi, WenyiZauderer, Marjorie G.Li, XiangZavattaro, ElisaLi, Xiang-GuoZaza, GianluigiLi, XiaohongZdarta, JakubLi, YanZebisch, ArminLi, YangZehbe, IngeborgLi, YinZeidán-Chuliá, FaresLi, ZhaohuiZeier, JürgenLiagre, BertrandZeilinger, CarstenLiang, Chi-TeZemel, MichaelLiang, QiangrongZenclussen, AnaLiao, Albert T.Zeng, HongmeiLiao, Francesca-FangZeng, HulieLiao, Jiahn-HaurZeppieri, MarcoLiao, Jyh-feiZeugolis, DimitriosLiao, Pei-ChunZgaga, LinaLiao, PengZhang, BoLiao, Yi-JenZhang, CaiguoLiapi, CharisZhang, ChiLibault, MarcZhang, DeqiangLiberale, LucaZhang, DianbaoLiby, KarenZhang, DuoLicausi, FrancescoZhang, HengyouLichtstein, DavidZhang, HuanminLiddell, Jeffrey R.Zhang, JianMinLidon, FernandoZhang, Jian-minLieberman, ScottZhang, JinnanLiebman, SusanZhang, JunranLiedtke, WolfgangZhang, KeLightfoot, AdamZhang, KezhongLigterink, WilcoZhang, LeiLim, BoraZhang, RuiyongLim, EdwinZhang, ShulingLim, Eun-KyungZhang, SuiLim, Jae HyangZhang, WeiLim, Ji-HongZhang, WeijieLim, JunxianZhang, WencaiLim, KhoonZhang, XiangLim, Kwang-ilZhang, XiaohuiLim, SoyeonZhang, XiaoyanLim, Sung-JigZhang, XumingLim, Sun-HyungZhang, YaliLim, Tae-GyuZhang, YeLim, YoonghoZhang, YingyueLima, RitaZhang, YixiangLin, Chien-hongZhang, YixuanLin, Chih-ChienZhang, Yuan-MingLin, Chih-JenZhang, YunkaiLin, Chih-LiZhang, ZhiyongLin, DingboZhang, ZhiyueLin, GiginZhao, BaoyinLin, Hai-ShuZhao, JichaoLin, HoZhao, LiangLin, Kwang-HueiZhao, MinLin, Liang-TzungZhao, NingningLin, Patrick P.Zhao, QiLin, Pei-HuiZhao, QingnanLin, ShengdaZhao, RuimingLin, Shih-YiZhao, ShanLin, WenshengZhao, SifengLin, YiguangZhao, WenxiuLin, Ying-HungZhao, ZongminLin, ZongtaoZheng, GuopingLinder, StigZheng, HuanquanLindquist, Diana M.Zheng, ShilongLindsay, Andrew J.Zheng, YueLindsay, HowardZhivkova, Zvetanka D.Lindsey-Boltz, LauraZhong, HaizhenLindstrom, Jon MartinZhong, JinshunLinge, AnnettZhong, TuhuaLingua, GuidoZhong, Wei-ZhuLinninger, Andreas A.Zhou, BangjunLino-Neto, TeresaZhou, BeiyanLionetti, VincenzoZhou, DezhongLionetto, Maria GiuliaZhou, GuangmingLipiński, SewerynZhou, Heshan SamLippens, GuyZhou, JiaLippert, BernhardZhou, LinLiLisec, JanZhou, ShuanhuLitchfield, DavidZhou, WenchaoLithgow, BrianZhou, Xue-RongLitofsky, Norman ScottZhou, Yi-hongLitt, AmyZhou, YubinLiu, AiminZhou, ZhichaoLiu, Cheuk LunZhou, ZhixiongLiu, Chia-ChiZhu, BingLiu, Chun-YuZhu, Guo-ZhangLiu, DelongZhu, HongyanLiu, FeiZhu, JingenLiu, Guang-YawZhu, QiuyuLiu, Guo JunZhu, WeiLiu, HaipeiZhu, XiangweiLiu, JiaZhu, XiaopingLiu, JianŽiarovská, JanaLiu, JianfengZiche, MarinaLiu, JunZichel, RanLiu, Jun-JenZiebell, HeikoLiu, JunliZiegler, PatrickLiu, RengyunZielenkiewicz, PiotrLiu, Shing-HwaZielińska-Pisklak, Monika A.Liu, TianboZima, AnetaLiu, WeiZimmer, GertLiu, WeizhenZimmer, MarcLiu, XiangshengZimmer, Warren E.Liu, YangZimmerberg, JoshuaLiu, YiZimmermann, HerbertLiu, Yi-ShiuanZloh, MireLiu, YongliangZmijewski, MichalLiu, YongqingZollner, ThomasLiu, YuZoltan-Istvan, SzaboLiu, ZhaohuiZorc, BrankaLiu, ZhihuiZordoky, BeshayLiu, ZhiqiuZou, QuanLiu, ZhixiaZsolnai, AttilaLiu-Smith, FengZubieta, ChloeLizardi-Mendoza, JaimeZugaza, J. L.Ljubimov, Alexander V.Žukauskaitė, AstaLjungdahl, PerZulli, A.Llorens, EugenioZüllig, RichardLloret, AnaZúñiga, SoniaLloyd, MatthewZuo, JianLluis, FredericZupko, IstvanLo Muzio, LorenzoZuppinger, ChristianLo, Amy C. Y.Zuvela, PetarLobaccaro, Jean-MarcZwack, PaulLoboda, AgnieszkaZweier, ChristianeLocascio, AnnamariaŻyżelewicz, DorotaLocatelli, Emanuele


